# Combination of HDAC inhibitor and PI3K inhibitor suppresses autophagy and induces apoptosis via cytoplasmic IκBα stabilization in p53-mutant diffuse large B-cell lymphoma

**DOI:** 10.1038/s41420-025-02756-7

**Published:** 2025-10-06

**Authors:** Jingwei Yao, Mengqi Li, Yuelong Jiang, Nanye Yao, Yating Liu, Liemei Lv, Yuchen Li, Jiewen Huang, Jie Zha, Bing Xu

**Affiliations:** 1 Department of Hematology, The First Affiliated Hospital of Xiamen University and Institute of Hematology, School of Medicine, Xiamen University, Xiamen, Fujian China; 2 Key Laboratory of Xiamen for Diagnosis and Treatment of Hematological Malignancy, Xiamen, Fujian China; 3 Lianjiang County Hospital, Fuzhou, Fujian China; 4 School of Pharmaceutical Sciences, Xiamen University, Xiamen, Fujian China

**Keywords:** B-cell lymphoma, Translational research, B-cell lymphoma

## Abstract

p53-mutant (p53-MUT) diffuse large B-cell lymphoma (p53^+^ DLBCL) remains a treatment-refractory DLBCL subtype lacking effective therapies. In this study, we systematically validated the synergistic therapeutic potential of HDAC inhibitor chidamide and PI3K inhibitor duvelisib in p53^+^ DLBCL through cellular models, in vivo experiments, and clinical samples. The combination regimen demonstrated robust induction of apoptosis across multiple p53^+^ DLBCL cell lines and primary clinical samples. Furthermore, it effectively reduced tumor burden in xenograft mouse models and prolonged overall survival. To elucidate the underlying mechanisms, clinical DLBCL tumor specimens from patients with p53-mutated and p53-wild-type genotypes, as well as p53^+^ DLBCL cell line samples before and after treatment with chidamide and duvelisib, were collected for RNA-seq analysis. Mechanistically, the combination stabilized IκBα via dual inhibition of PI3Kδ and HDAC2, thereby suppressing NF-κB-p65 phosphorylation and subsequent nuclear translocation, concurrently inhibiting autophagy. These pathway disruptions collectively led to tumor proliferation arrest and potentiation of apoptosis. Specifically, duvelisib inhibited IKK phosphorylation to prevent IκBα degradation, while chidamide enhanced acetylation of histone H1.5 by targeting lysine residues at positions K67 and K93. This acetylation promoted histone H1.5-IκBα interactions, further stabilizing IκBα and attenuating p65 nuclear trafficking. Our findings identify a novel and potent therapeutic strategy for p53^+^ DLBCL, warranting clinical translation.

## Introduction

Diffuse large B-cell lymphoma (DLBCL), an aggressive form of non-Hodgkin lymphoma (NHL), accounts for 30–40% of all NHL cases and demonstrates rapid disease progression [1–3]. While the R-CHOP regimen remains the first-line therapeutic approach for DLBCL, approximately 30% of patients exhibit poor response to frontline treatment, particularly those with TP53-mutated DLBCL or relapsed disease [4, 5]. A retrospective analysis of 506 DLBCL cases revealed that patients with p53 mutations exhibited significantly shorter median overall survival (OS) compared to those with wild-type p53 [6]. Furthermore, under the R-CHOP immunochemotherapy regimen, the 3-year OS rate remained substantially lower in the p53-mutated cohort than in wild-type counterparts (65.1% vs 80.6%, respectively) [7]. The compromised efficacy of R-CHOP in TP53-mutated cases may stem from impaired DNA damage response and apoptosis resistance mediated by p53 dysfunction. Concomitantly, p53 mutations confer resistance to conventional immunochemotherapeutic regimens, necessitating novel therapeutic strategies [6, 7].

In diffuse large B-cell lymphoma (DLBCL), constitutive activation of the NF-κB signaling pathway and enhanced cellular stress tolerance are frequently observed, collectively contributing to tumor cell survival and chemoresistance [8]. Notably, p53 mutations are commonly associated with augmented NF-κB transcriptional activity [9]. The NF-κB family comprises a group of ubiquitously expressed transcription factors that play pivotal roles in cell survival regulation through apoptosis inhibition during chemotherapeutic responses [9]. This transcription factor family consists of five structurally related members: RelA (p65), RelB, c-Rel, NF-κB1 (p50 and its precursor p105), and NF-κB2 (p52 and its precursor p100). Under basal conditions, these proteins remain sequestered in the cytoplasm through interaction with inhibitory IκB proteins, predominantly IκBα. Upon cellular stimulation, NF-κB members dissociate from IκBα and translocate to the nucleus to activate downstream target genes [10, 11]. Emerging evidence demonstrates that IκBα not only regulates NF-κB signaling but also modulates p53 functional activity. The protein interacts with wild-type p53 to regulate tumor cell apoptosis [12]. In p53-mutated prostate cancer models, NF-κB inhibition induces phosphorylation of mutant p53 at serine 15 (Ser15), leading to enhanced protein stabilization and subsequent apoptosis. Conversely, IκB overexpression promotes phosphorylation of mutant p53 and potentiates apoptotic cell death [9].

Notably, p53 mutations not only influence NF-κB pathway activity and IκBα function but also exhibit extensive crosstalk with autophagy-related pathways [13]. The autophagic pathway may reduce the autophagic degradation of mutant p53 proteins, ultimately contributing to tumor progression [14]. As a classical pro-inflammatory transcription factor, NF-κB regulates not only cell survival and immune responses but also modulates autophagic processes [15]. Under stress conditions, NF-κB-mediated IL-6/STAT3 signaling promotes enhanced autophagy in mantle cell lymphoma (MCL), thereby supporting malignant cell survival [15]. Therefore, targeting the regulation of NF-κB and autophagy may represent a critical therapeutic strategy for refractory DLBCL.

Histone deacetylases (HDACs) regulate chromatin architecture and gene expression through histone modification, critically influencing cellular processes including proliferation, differentiation, and apoptosis [16, 17]. Chidamide is a novel benzamide-type histone deacetylase (HDAC) inhibitor [18–21]. Recent clinical trials confirm its favorable safety profile and therapeutic efficacy through histone hyperacetylation-mediated tumor suppressor gene reactivation [22]. Nevertheless, its biological impact in TP53-mutated DLBCL remains undefined.

The phosphoinositide 3-kinase (PI3K) family regulates critical oncogenic processes, including cell survival and metabolism, with PI3K pathway hyperactivation being particularly prevalent in hematological malignancies [23]. Duvelisib, a dual PI3K-δ/γ inhibitor, demonstrates clinical activity in relapsed/refractory B-cell lymphomas, though adaptive resistance frequently limits durable responses [24–26]. Mechanistically, duvelisib exerts p53-independent cytotoxicity through direct proliferation inhibition and apoptosis induction [27]. Although HDAC/PI3K inhibitor monotherapy has demonstrated partial clinical activity in early-phase trials, accumulating clinical evidence suggests that targeting a single oncoprotein often proves inadequate to achieve desired therapeutic outcomes due to functional redundancy in alternative pathways or acquired resistance mutations [28, 29], which ultimately enable tumor cell survival and therapeutic evasion. Guided by this mechanistic rationale, the present study proposes a dual-targeting therapeutic strategy that sustains high efficacy even under p53-mutated conditions. This innovative strategy aims to overcome the clinical challenges associated with TP53-mutated DLBCL by establishing a complementary cytotoxic mechanism, thereby potentially improving patient prognosis and providing a novel strategy for precision treatment.

Our findings demonstrate that chidamide-duvelisib coadministration significantly suppresses autophagy and enhances apoptosis in TP53-mutated DLBCL cells, with consistent efficacy observed in vivo and clinical samples. Mechanistically, duvelisib and chidamide exhibit synergistic antitumor activity through coordinated suppression of NF-κB-p65 nuclear translocation. This cooperative effect is achieved via dual targeting of PI3K-δ and HDAC2, respectively, resulting in enhanced stabilization of IκBα protein. These results position chidamide-duvelisib combination therapy as a promising strategy for TP53-mutated DLBCL, potentially addressing critical unmet clinical needs in this high-risk population.

## Result

### The combination of chidamide and duvelisib significantly promotes apoptosis and inhibits proliferation in p53^+^ DLBCL cells

**Fig. 1:**
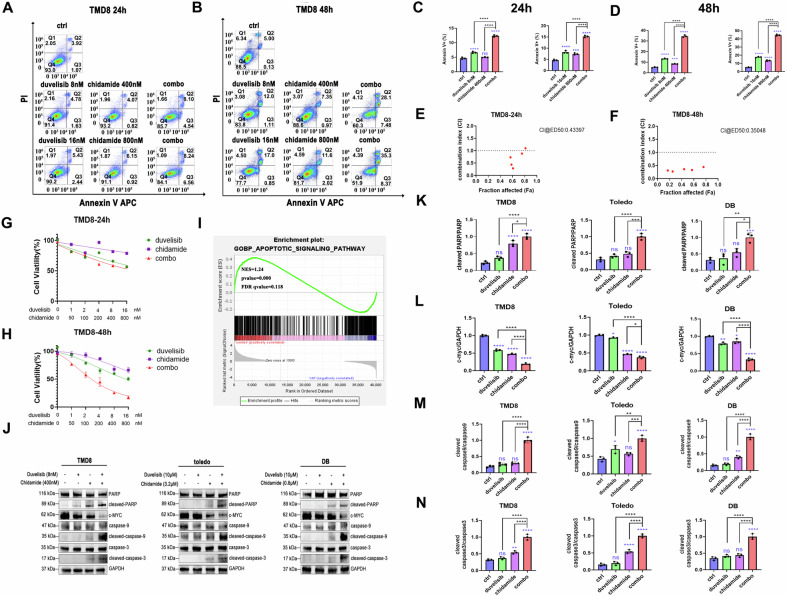
Chidamide and duvelisib jointly enhance apoptosis and reduce proliferation in p53^+^ DLBCL cells. **A–D** TMD8 cells were treated with chidamide, duvelisib, or their combination at indicated concentrations for 24/48 h. Apoptosis was quantified by Annexin V/PI flow cytometry (apoptotic cells defined as Annexin V-positive). Data represent mean ± SD of triplicate experiments. Statistical significance (**P < 0.01, ***P < 0.001, ****P < 0.0001; ns) was analyzed by one-way ANOVA. **E–H** TMD8 cells were treated with indicated concentrations of chidamide and duvelisib for 24 or 48 h, respectively. The inhibitory effects on cell viability were subsequently assessed using the CCK-8 assay kit. Synergistic, additive, or antagonistic interactions between the two agents were quantitatively analyzed by calculating combination index (CI) values via CompuSyn software, in accordance with the Chou-Talalay method. **I** Gene Set Enrichment Analysis demonstrated significant enrichment of apoptotic signaling pathways following pharmacological treatment. **J–N** Following 24-hour treatment with chidamide and duvelisib in TMD8, Toledo, and DB cell lines, western blotting analysis was performed to evaluate the expression levels of apoptosis-related and tumor-associated proteins. The pharmacological interventions significantly upregulated key apoptotic markers, including cleaved-caspase3, cleaved-caspase9, and cleaved-PARP. Concurrently, a notable downregulation was observed in the oncogenic protein c-myc expression. In cell-based assays and western blotting, DMSO served as the vehicle control, diluted in culture medium to a final concentration of 0.05% (v/v).

To evaluate the therapeutic effects of chidamide and duvelisib, apoptosis in three p53^+^ DLBCL cell lines (TMD, Toledo, and DB) was analyzed using Annexin V/PI dual staining followed by flow cytometry after 24 h and 48 h of drug exposure. Both chidamide and duvelisib monotherapy induced apoptosis in p53-mutated DLBCL cells, while the combination regimen demonstrated significantly enhanced efficacy compared to either agent alone. Consistent results were observed at both 24 h and 48 h timepoints (Fig. 1A–D**;** Supplementary Figs. 1, 2). Subsequently, the anti-proliferative effects of these agents were assessed using the Cell Counting Kit-8 (CCK-8) assay. Dose- and time-dependent inhibition of cell proliferation was observed following treatment with chidamide or duvelisib, with the combination therapy exhibiting superior inhibitory effects relative to single-agent treatments (Fig. 1E–H; Supplementary Figs. 1, 2). Subsequently, we investigated the efficacy of duvelisib combined with chidamide in p53 wild-type (p53-WT) DLBCL. Validation was performed using three p53-WT cell lines, and the results demonstrated that the duvelisib-chidamide combination also exerted a favorable synergistic effect in p53-WT DLBCL (Supplementary Figs. 3–5). Intriguingly, after 48 h of treatment, the combined effect was more pronounced in p53-mutant DLBCL compared to p53-WT DLBCL (Supplementary Fig. 5I, J).

To further validate these findings, RNA sequencing (RNA-seq) combined with Gene Set Enrichment Analysis (GSEA) was performed on pre- and post-treatment cells. The combination therapy demonstrated significant positive enrichment of apoptosis-related pathways (*p* < 0.001, FDR < 0.25; Fig. 1I). Immunoblotting analysis revealed that combined chidamide and duvelisib treatment markedly upregulated the expression of apoptotic markers, including cleaved caspase-3, cleaved caspase-9, and cleaved PARP, while downregulating the oncoprotein c-My**c**(Fig. 1J–N). Collectively, these comprehensive in vitro experiments demonstrate that the chidamide-duvelisib combination synergistically enhances apoptosis and suppresses proliferation in p53^+^ DLBCL cells.

### Combined treatment of chidamide and duvelisib suppresses tumor growth in p53-mutant DLBCL xenograft models

**Fig. 2:**
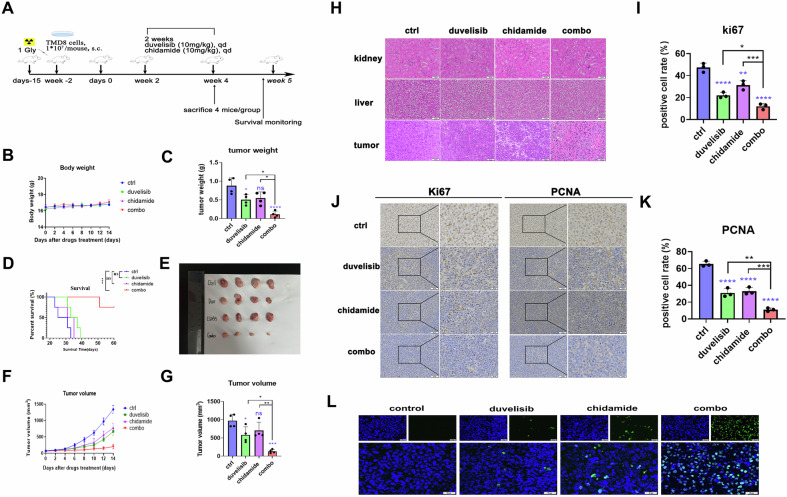
Combination of chidamide and duvelisib inhibits tumor growth in a p53-mutant DLBCL xenograft model. A p53^+^ DLBCL tumor xenograft mouse model was established using TMD8 cells (**A**). Daily oral gavage administration with chidamide, duvelisib, or their combination was initiated post-tumor formation. Mouse body weight (**B**) and tumor volume (**F**) were measured daily. **E** Subcutaneous tumors were excised and photographed at experimental endpoint, with intergroup comparisons of tumor weight (**C**) and volume (**G**) for efficacy evaluation. Liver, kidney, and tumor tissues were fixed for hematoxylin-eosin (HE) staining(**H**). Immunohistochemical detection of Ki67 and PCNA expression was performed to assess tumor cell proliferation in vivo (**I**–**K**). Tumor cell apoptosis was evaluated by TUNEL staining (**L**). Kaplan-Meier overall survival (OS) curve of xenograft mice (**D**). Data are presented as mean ± SD analyzed by one-way ANOVA, with *p < 0.05, **p < 0.01, ***p < 0.001, and ****p < 0.0001.

To further validate our findings in vivo, we established p53^+^ DLBCL tumor xenograft mouse models using TMD8 cells (Fig. 2A). Treatment regimens demonstrated no significant impact on murine body weight (Fig. 2B). The combination of duvelisib and chidamide significantly inhibited tumor volume and weight in mice, with markedly superior efficacy compared to monotherapy alone (Fig. 2C, E–G).

Tissue specimens, including liver, kidney, and tumor tissue,s underwent comprehensive histopathological evaluation through H&E staining, immunohistochemistry (IHC), and TUNEL apoptosis assays. The results revealed no apparent hepatorenal toxicity across treatment groups (Fig. 2H). ELISA quantification of serum IL-6 and IL-17 levels in mice revealed that duvelisib monotherapy induced a marginal increase in IL-6, whereas IL-17 levels remained unaltered. However, co-administration of chidamide with duvelisib resulted in no significant alterations in either IL-6 or IL-17 compared to controls. Collectively, these data suggest that the combined immunotoxicity profile of chidamide and duvelisib falls within acceptable limits (Supplementary Fig. 6). The combination therapy group demonstrated significant suppression of tumor cell proliferation and progression (Fig. 2H–L). The combination therapy group showed significantly prolonged survival compared to both control and monotherapy groups (Fig. 2D). These collective findings strongly suggest synergistic antitumor efficacy of chidamide-duvelisib coadministration in p53-mutant DLBCL models.

### Chidamide combined with duvelisib promotes apoptosis by suppressing autophagy in p53^+^ DLBCL

**Fig. 3:**
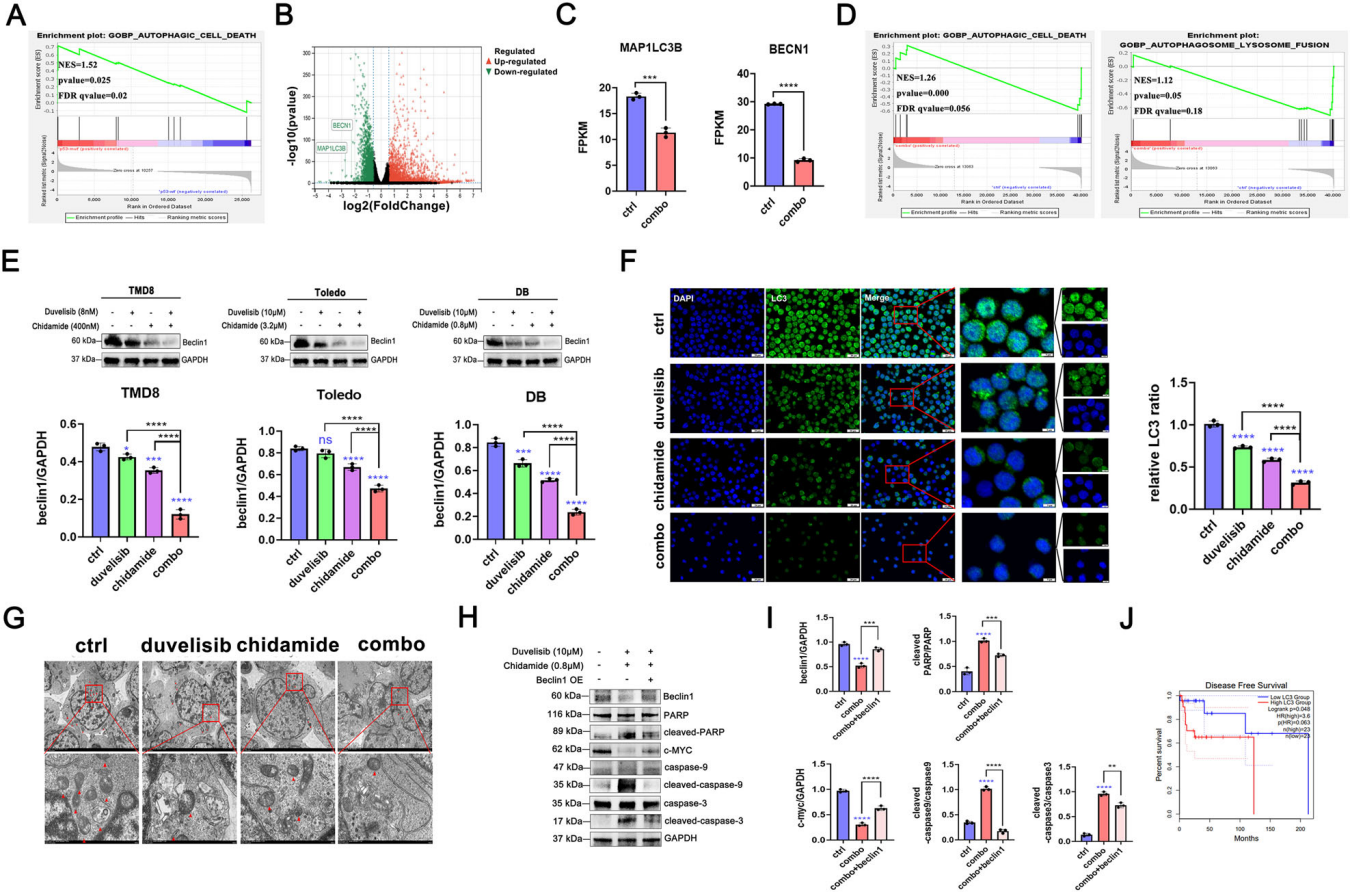
In p53^+^ DLBCL, combined chidamide and duvelisib induce apoptosis by suppressing autophagy. Tumor specimens from 6 DLBCL cases with p53-mutant and 6 cases with p53-wild-type were collected. RNA-seq was performed to profile transcriptomes, followed by gene set enrichment analysis (GSEA) for identifying signaling pathway alterations (**A**). DB cells were exposed to specified concentrations of chidamide, duvelisib, or their combination for 24 hours, followed by RNA sequencing. DEGs were screened (**B**, **C**) and subjected to enrichment analysis (**D**). Western blotting was performed to detect the expression of the autophagy-related protein Beclin1 in TMD8, Toledo, and DB cells post-treatment (**E**). Immunofluorescence was utilized to assess expression levels of the autophagic marker LC3 (**F**). TEM was employed to examine alterations in autolysosomes following drug treatment (**G**). A DB-Beclin1-overexpressing cell line was constructed for further validation, wherein the apoptosis process originally promoted by combination therapy was partially rescued in Beclin1-overexpressing cells (**H**, **I)**. The GEPIA database was analyzed to evaluate the prognostic relationship between LC3 expression and survival outcomes in DLBCL patients (**J**). In cell-based assays and western blotting, DMSO served as the vehicle control, diluted in culture medium to a final concentration of 0.05% (v/v).

We obtained tumor specimens from 6 DLBCL patients with TP53 mutations (Sanger sequencing-confirmed) and 6 wild-type TP53 controls (total *n* = 12, Supplementary Table 2). RNA sequencing integrated with Gene Set Enrichment Analysis (GSEA) revealed significant enrichment of the autophagy pathway in the mutant cohort compared to wild-type (Fig. 3A, Supplementary Fig. 7A–C, E). Consequently, we subsequently treated p53-mutant DLBCL cells with chidamide and duvelisib and performed post-treatment RNA-seq analysis. The results revealed significant downregulation of autophagy-related genes LC3 and beclin1 following combined treatment with chidamide and duvelisib (Fig. 3B, C). Gene expression profiles were interrogated through GSEA to further explore the relationship between therapeutic interventions and autophagy-related pathways. Notably, the analysis demonstrated significant negative enrichment of autophagy-related pathways post-combination therapy (*p* < 0.001, FDR < 0.25; Fig. 3D). To validate these findings, we examined autophagy dynamics in p53^+^ DLBCL cells. Western blotting showed that the combination treatment markedly reduced the expression of beclin1, a hallmark autophagy-related protein (Fig. 3E). Similarly, immunofluorescence analysis revealed substantial inhibition of LC3 expression, indicating suppressed autophagic activity (Fig. 3F). Electron microscopy further confirmed a significant reduction in autolysosome formation, consistent with impaired autophagy progression (Fig. 3G). These results collectively demonstrate robust suppression of autophagy following combination therapy. To investigate whether autophagy inhibition directly contributes to apoptosis induction, we established a DB-beclin1-overexpressing cell line (Supplementary Fig. 8A). Notably, the pro-apoptotic effect of the combination therapy was partially rescued in beclin1-overexpressing cells (Fig. 3H, I), suggesting that autophagy suppression plays a critical role in promoting apoptosis. Finally, survival analysis using the GEPIA database revealed that low LC3 expression correlated with improved prognosis in DLBCL patients (Fig. 3J), aligning with our experimental findings.

### Chidamide combined with duvelisib suppresses autophagy and promotes apoptosis by inhibiting nuclear translocation and phosphorylation of NF-κB p65

**Fig. 4:**
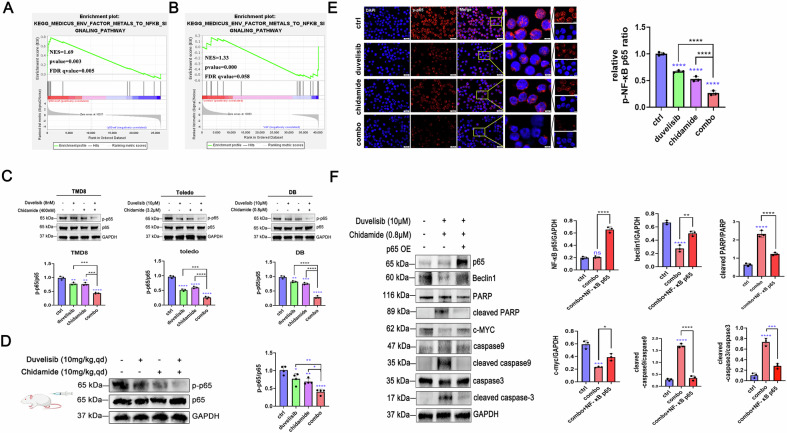
Chidamide and duvelisib synergistically inhibit autophagy and promote apoptosis by suppressing NF-κB p65 nuclear phosphorylation. **A** Tumor tissues from 12 DLBCL patients (6 TP53-mutant and 6 TP53-wild-type) underwent RNA sequencing. GSEA was applied to identify dysregulated signaling pathways. **B** RNA-seq results revealed that after 24 h drug exposure in DB cells, DEGs were significantly enriched in the NF-κB pathway. **C**, **D** Western blotting analysis was performed to detect the expression levels of NF-κB p65 and phosphorylated NF-κB p65 (Ser536) (p-NF-κB p65) in TMD8, Toledo, DB cells and p53-mutant DLBCL Xenograft Models Tumor Tissue post-drug treatment. **E** Immunofluorescence staining was employed to assess the expression of p-NF-κB p65 in drug-treated cells. **F** In established DB-p65 overexpression cell models, compared with the duvelisib and chidamide combination therapy group, p65-overexpressing cells exhibited partial restoration of downregulated Beclin1 levels and significant attenuation of drug-induced apoptotic progression when treated with the same drug combination. In cell-based assays and western blotting, DMSO served as the vehicle control, diluted in culture medium to a final concentration of 0.05% (v/v).

Comparative analysis of clinical specimens from p53-mutated DLBCL patients versus p53 wild-type DLBCL cases revealed a significant enrichment of the NF-κB signaling pathway in the p53-mutant subgroup (Fig. 4A, Supplementary Fig. 7D). Subsequent GSEA validation of drug-treated cells further revealed marked negative enrichment of the NF-κB signaling pathway following combination therapy (*p* < 0.001, FDR < 0.25; Fig. 4B). To validate these findings, we examined NF-κB expression changes and their functional impact on autophagy and apoptosis. Western blotting analysis confirmed that the expression of phosphorylated NF-κB p65 was significantly reduced post-treatment. Compared to chidamide or duvelisib monotherapy, the combination regimen demonstrated enhanced inhibitory efficacy, as evidenced by more pronounced suppression of NF-κB pathway activation. These consistent findings were observed across both p53-mutated DLBCL cell lines and corresponding xenograft models, suggesting a synergistic therapeutic effect of the combined intervention **(**Fig. 4C, D**)**. Immunofluorescence staining of p-NF-κB p65 corroborated these results, demonstrating diminished expression in the combination group **(**Fig. 4E**)**.

To assess the mechanistic role of NF-κB p65 suppression, we constructed a p65-overexpressing p53^+^ DLBCL cell line (Supplementary Fig. 8B). In these cells, p65 overexpression partially rescued the downregulation of Beclin1 and attenuated the apoptosis-promoting effects induced by chidamide and duvelisib combination treatment (Fig. 4F). These results indicate that the suppression of autophagy and enhancement of apoptosis by the drug combination are mediated through inhibition of NF-κB p65 activity. Collectively, our data demonstrate that chidamide synergizes with duvelisib to inhibit nuclear translocation and phosphorylation of NF-κB p65, thereby blocking autophagy and driving apoptosis.

### Duvelisib and chidamide stabilize IκBα in the cytoplasm through selective targeting of PI3K delta and HDAC2

**Fig. 5:**
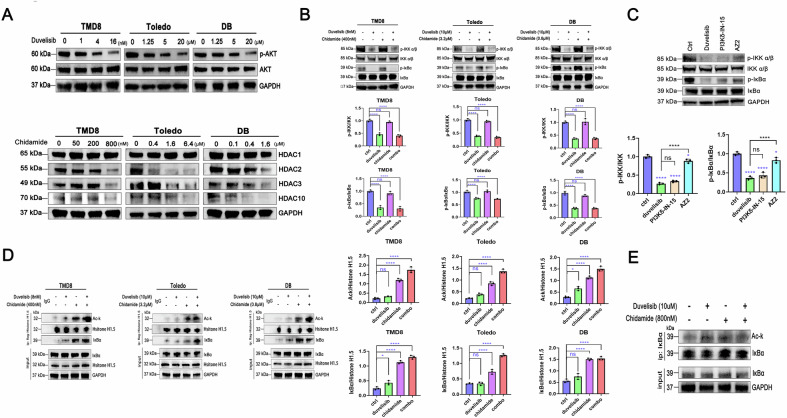
Duvelisib and chidamide stabilize IκBα via PI3Kδ and HDAC2 targeting, respectively. **A** Western blotting analysis was performed to determine the expression levels of drug-targeted proteins corresponding to chidamide (HDAC1, HDAC2, HDAC3, HDAC10) and duvelisib (AKT, p-AKT Ser473) in TMD8, Toledo, and DB cells following gradient concentration treatment with chidamide, duvelisib, or their combination. **B** Western blotting was utilized to assess the protein expression levels of IKKα/β, p-IKKα/β (Ser176/180), IκBα and p-IκBα (Ser32) in DB cells after treatment with specified drug concentrations. **C** The effects of duvelisib, PI3Kδ inhibitor (PI3Kδ-IN-15), and PI3Kγ inhibitor (AZ2) on IKK and IκBα protein expression in DB cells were evaluated by western blotting. **D** Following histone H1.5 protein recruitment, the acetylation status of histone H1.5 and its interaction with IκBα were examined. **E** A co-immunoprecipitation (co-IP) assay was employed to directly detect the acetylation level of IκBα. In western blotting, DMSO served as the vehicle control, diluted in culture medium to a final concentration of 0.05% (v/v).

We first validated the on-target specificity of chidamide and duvelisib in p53-mutated DLBCL models, confirming the absence of off-target effects for both agents. As demonstrated in Fig. 5A, duvelisib inhibited p-AKT expression across three p53-mutated DLBCL cell lines, whereas chidamide exhibited dose-dependent suppression of HDAC2, HDAC3, and HDAC10 without modulating HDAC1 expression.

In the canonical NF-κB signaling cascade, IκBα suppresses the transcriptional activity of the p65/p50 heterodimer by binding to the complex in the cytoplasm, where IκBα itself remains stabilized in a non-phosphorylated state. Extracellular stimuli activate IKK complexes, which phosphorylate IκBα and induce its degradation. This process liberates NF-κB p65 for nuclear translocation and subsequent transcriptional activation. To delineate the regulatory roles of duvelisib and chidamide in this pathway, we examined IKK and IκBα protein dynamics following single or combined treatments. Figure 5B reveals that duvelisib substantially attenuated phosphorylation of both IKK and IκBα (with greater potency against IκBα), while chidamide showed negligible effects. These data imply that duvelisib stabilizes IκBα by blocking its phosphorylation, thereby reducing NF-κB p65 dissociation. Subsequent validation using isoform-selective PI3K inhibitors established that PI3K-δ inhibition constitutes the primary mechanism underlying duvelisib-mediated IκBα stabilization (Fig. 5C).

Notably, our prior western blotting analysis (Fig. 4C) suggested chidamide contributes to attenuated p65 dissociation. To interrogate this mechanism, we conducted comparative mass spectrometry of control versus chidamide-treated groups. Proteomic profiling revealed a pronounced interaction between histone H1.5 and IκBα, coupled with a marked increase in histone H1.5 acetylation upon chidamide treatment (Table 1). We postulated that chidamide-induced hyperacetylation of histone H1.5 enhances its binding to IκBα, thereby stabilizing IκBα and reducing p65 liberation. Immunoprecipitation assays corroborated this hypothesis, showing elevated histone H1.5 acetylation and strengthened histone H1.5–IκBα interaction in chidamide-treated samples (Fig. 5D). Crucially, direct assessment of IκBα acetylation status revealed no alterations (Fig. 5E), excluding a cell-autonomous acetylation mechanism for IκBα. Collectively, these findings indicate that chidamide stabilizes IκBα through a non-canonical pathway involving histone H1.5 hyperacetylation and subsequent reinforcement of histone H1.5–IκBα binding. To determine whether mutant p53 plays a role in regulating IκBα stability during treatment, we analyzed p53 expression before and after drug administration. Interestingly, we observed no significant changes in p53 levels, providing additional evidence that the combination therapy achieves its effects through p53-independent pathways (Supplementary Fig. 9).

**Table 1:** Mass spectrometry-based detection of differential acetylation in Histone H1.5 between chidamide-treated and control groups.

Accession	Unique	-10LgP	Start	End	AScore
P16401 | H15_HUMAN	N	49.02	94	113	K7:Methylation(KR):0.88;K16:Acetylation (K):0.88
P16401 | H15_HUMAN	N	47.29	101	122	K9:Acetylation (K):0.00;K13:Acetylation (K):0.00
P16401 | H15_HUMAN	N	90.7	89	100	K5:Acetylation (K):115.21
P16401 | H15_HUMAN	Y	69.16	37	49	K1:Acetylation (K):89.67
P16401 | H15_HUMAN	Y	58.47	67	82	K1:Acetylation (K):81.27
P16401 | H15_HUMAN	N	69.75	101	122	K12:Acetylation (K):0.00;K13:Methylation(KR):0.00
P16401 | H15_HUMAN	Y	31.41	37	49	K1:Formylation:54.32;)1:Acetylation (K):54.32
P16401 | H15_HUMAN	Y	32.43	37	49	K1:Dihydroxy:12.42;K13:Acetylation (K):22.74
P16401 | H15_HUMAN	Y	44.55	125	140	K6:Acetylation (K):5.99;K8:Methylation(KR):5.99
P16401 | H15_HUMAN	N	48.02	114	125	K9:Acetylation (K):5.08;K11:Acetylation (K):9.34
P16401 | H15_HUMAN	N	57.22	101	122	K12:Acetylation (K):0.00;K13:Acetylation (K):0.00
P16401 | H15_HUMAN	N	36.14	85	100	K4:Acetylation (K):23.70

### Chidamide treatment suppresses HDAC2 activity, inducing histone H1.5 acetylation and modulating IκBα-p65 signaling

**Fig. 6:**
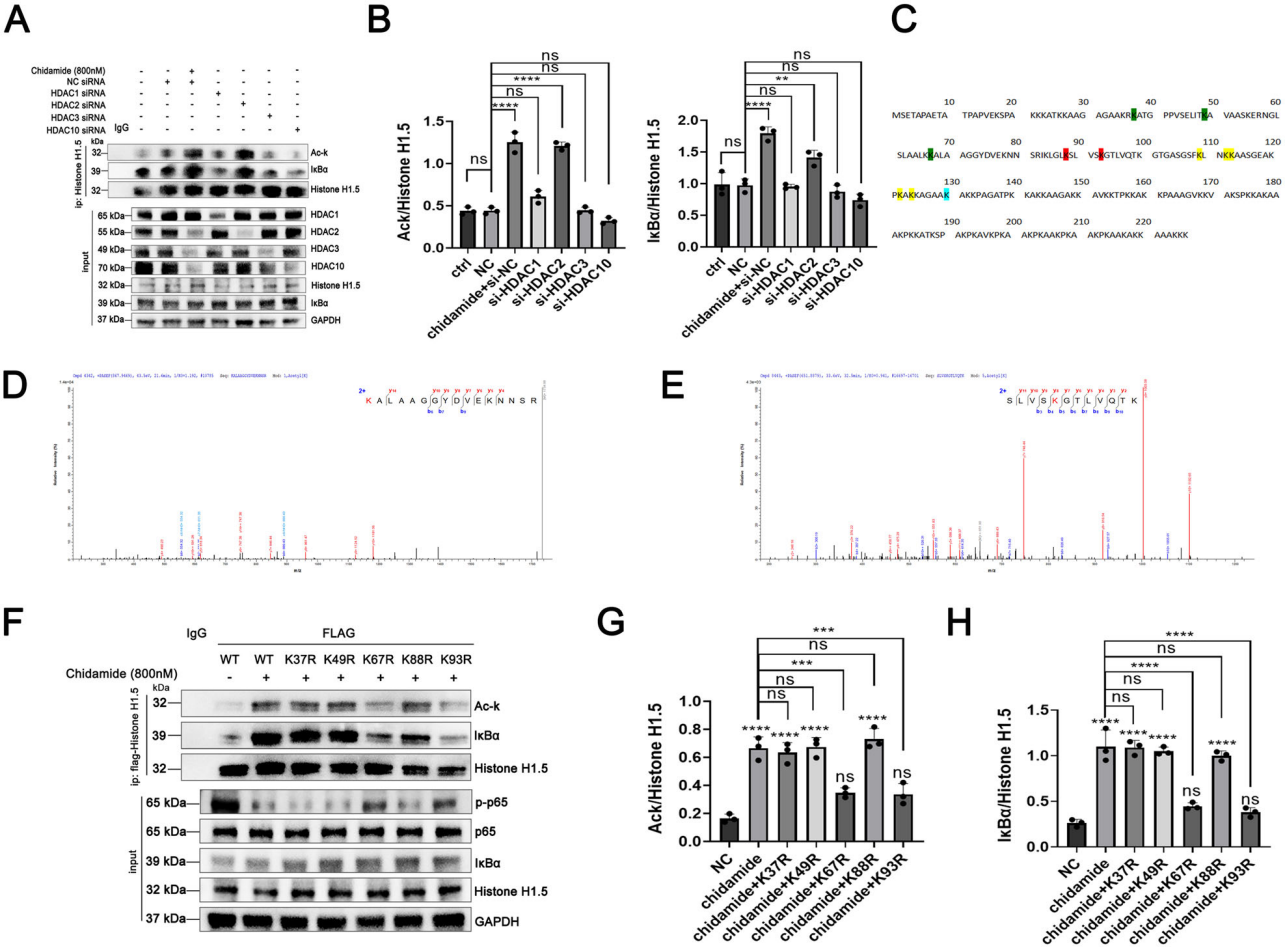
Chidamide treatment inhibits HDAC2 activity and leads to histone H1.5 acetylation. **A**, **B** Knockdown of HDAC1, HDAC2, HDAC3, and HDAC10 was performed using siRNA. Following histone H1.5 enrichment, western blotting analysis was conducted to detect histone H1.5 acetylation levels and its interaction with IκBα. (**C**) Mass spectrometry identified five histone H1.5 acetylation sites (A-score >13) after chidamide treatment. **D**–**H** Acetylation-deficient mutants were generated by replacing these lysine residues with arginine. Following histone H1.5 enrichment, co-immunoprecipitation (Co-IP) assays were performed to evaluate histone H1.5 acetylation, NF-κB p65 protein levels, and histone H1.5-IκBα interaction. The K67 and K93 mutations significantly reduced acetylation and weakened the histone H1.5-IκBα interaction.

To further identify the specific target through which chidamide promotes histone H1.5 acetylation, siRNA-mediated knockdown of HDAC1, HDAC2, HDAC3, and HDAC10 was performed, followed by immunoprecipitation of histone H1.5 and western blotting analysis. As shown in Fig. 6A, B, chidamide primarily enhanced histone H1.5 acetylation and its interaction with IκBα by inhibiting HDAC2, rather than other known chidamide targets.

Subsequently, mass spectrometry identified eight lysine residues on histone H1.5 exhibiting altered acetylation levels following chidamide treatment (Table 1; Fig. 6C). Among these, five sites achieved an A-score >13, including three specific and two non-specific peptides (Fig. 6D, E, Supplementary Fig. 10). To validate these findings, lysine-to-arginine (K-to-R) acetylation-defective mutants were generated at the five candidate residues (K37R, K49R, K67R, K88R, and K93R). Consistent with prior observations, chidamide treatment markedly elevated histone H1.5 acetylation levels. However, individual mutations at K67 and K93 significantly attenuated acetylation (Fig. 6F, G) and weakened the interaction between histone H1.5 and IκBα (Fig. 6F and H).

Notably, chidamide treatment suppressed phosphorylation of NF-κB p65 (p-p65), but this inhibitory effect was partially rescued in cells expressing the K67R or K93R mutants (Fig. 6F). These results collectively demonstrate that chidamide selectively inhibits HDAC2, thereby disrupting deacetylation at K67 and K93 of histone H1.5. This leads to hyperacetylation of histone H1.5, stabilization of its binding to IκBα, and subsequent suppression of p65 activation. The findings underscore a mechanistic link between HDAC2 inhibition, histone H1.5 acetylation dynamics, and modulation of the IκBα-p65 signaling axis.

### Synergistic targeting of p53-mutated DLBCL primary cells by duvelisib combined with chidamide

**Fig. 7:**
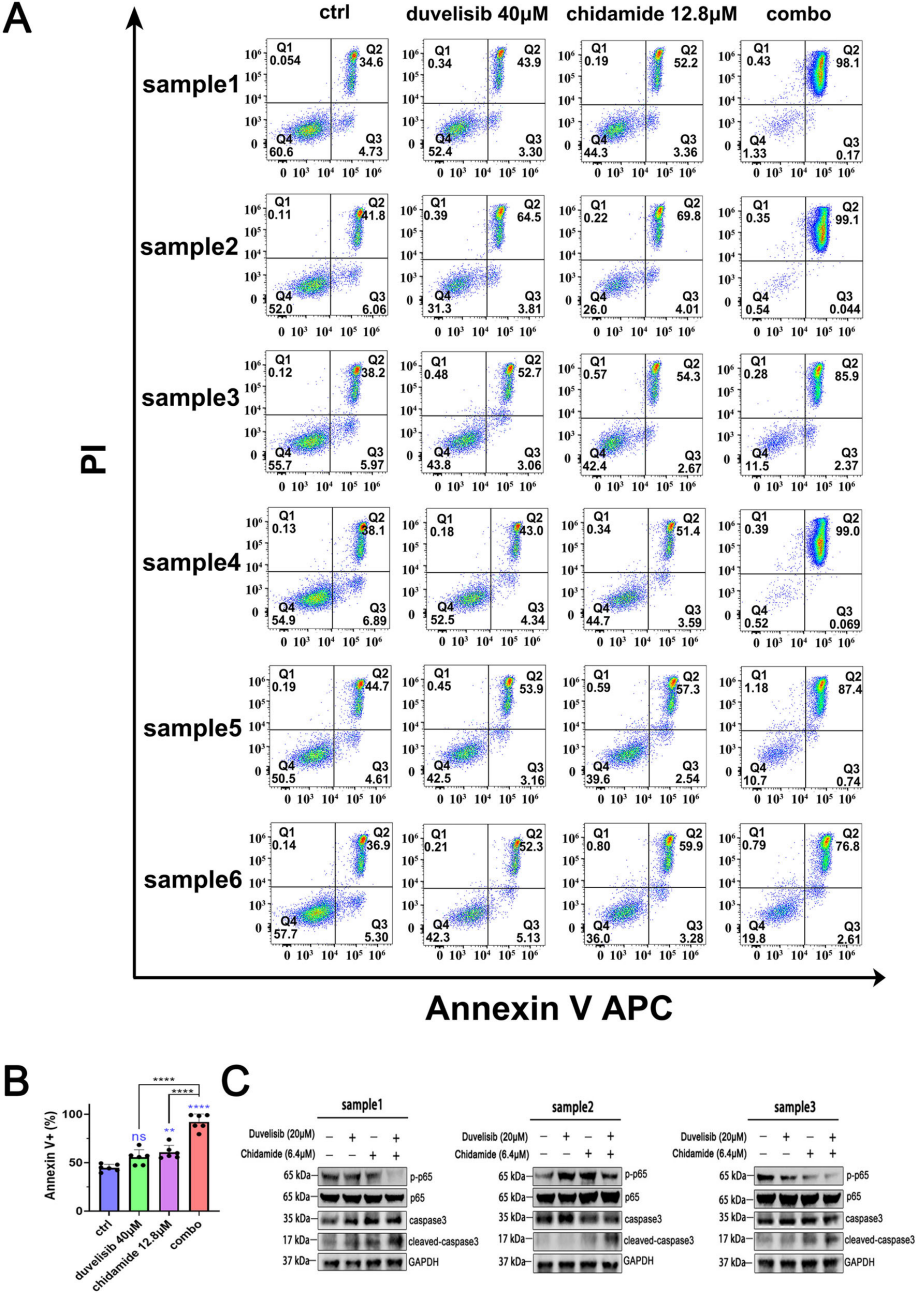
The combined administration of chidamide with duvelisib significantly induced apoptosis in primary DLBCL cells harboring p53 mutations and effectively suppressed NF-κB p65 nuclear translocation. **A**, **B** Primary DLBCL cells with p53 mutations were treated with indicated concentrations of chidamide and duvelisib for 24 h. Apoptosis was quantified by Annexin V/PI flow cytometry, with apoptotic cells defined as Annexin V^+^ populations. **C** The caspase3 and NF-κB p65 expression were analyzed by western blotting. In cell-based assays and western blotting, DMSO served as the vehicle control, diluted in culture medium to a final concentration of 0.05% (v/v).

Finally, to investigate the clinical applicability of chidamide combined with duvelisib, primary lymphoma cells were isolated from lymph node tissues of 6 patients with p53-mutated DLBCL and 6 patients with non-p53-mutated DLBCL as described in earlier sections. After a 24-hour treatment with chidamide, duvelisib, or their combination, both single agents induced apoptosis in primary cells, whereas the combination therapy exhibited a potent synergistic cytotoxic effect. This effect was consistently observed in both p53-mutated and p53-wild-type samples (Fig. 7, Supplementary Fig. 11). Western blotting analysis further demonstrated that the chidamide-duvelisib combination significantly suppressed nuclear translocation of NF-κB p65 in these p53-mutated primary DLBCL cells (Fig. 7C). These results collectively validate the therapeutic potential of dual-targeting strategies in clinically relevant p53-mutated DLBCL.

## Discussion

DLBCL with p53 mutation represents one of the most treatment-refractory subtypes in current clinical practice, posing significant therapeutic challenges in lymphoma management. The inherent aggressive behavior and enhanced drug resistance associated with p53-mutated DLBCL frequently lead to suboptimal response to conventional R-CHOP regimens. Furthermore, the current therapeutic landscape lacks effective targeted approaches specifically addressing this molecular subset. These clinical realities underscore the critical necessity for developing innovative treatment strategies to improve clinical outcomes in this high-risk patient population.

Recent studies demonstrate that tumor cells dynamically regulate key tumor suppressor gene silencing and oncogene activation through epigenetic reprogramming (e.g., aberrant DNA methylation or histone modification imbalance), thereby driving malignant phenotypes [30, 31]. Epigenetic mechanisms can directly intervene in cell cycle progression and apoptosis evasion through chromatin remodeling or non-coding RNA networks [32]. Targeting epigenetic nodes has emerged as a promising direction for precision oncology [33]. In DLBCL, previous clinicopathological investigations have revealed that elevated nuclear expression of histone deacetylases (HDACs), particularly HDAC2, may epigenetically regulate clinical outcomes and demonstrate a significant correlation with shortened survival duration [34]. Chidamide, a subtype-selective oral HDAC inhibitor approved for relapsed/refractory peripheral T-cell lymphoma (PTCL), exhibits specific targeting of HDAC1/2/3/10. This agent mediates epigenetic modulation to induce tumor cell cycle arrest, differentiation, and activation of non-canonical apoptotic signaling pathways, with its therapeutic effects being independent of the canonical p53 signaling pathway [35]. Our study further demonstrates that Chidamide selectively targets HDAC2 as a pivotal mechanism, ultimately leading to potent induction of tumor cell apoptosis. Although chidamide demonstrates moderate efficacy in PTCL (ORR: 28%, CR: 14%, median PFS: 2.1 months, OS: 21.4 months), its clinical utility is limited by hematologic toxicities, such as thrombocytopenia and neutropenia.

Notably, Duvelisib—a clinically available and commercially accessible dual PI3Kδ/γ inhibitor—has shown substantial antitumor efficacy in indolent non-Hodgkin lymphoma (iNHL), yet its broader clinical implementation is hampered by immune-related toxicities and infection risks [36, 37]. Mechanistically, the combination therapy of chidamide and duvelisib exploits their complementary pharmacological actions: HDAC inhibition drives epigenetic reprogramming to prime lymphoma cells for apoptosis, while PI3Kδ/γ blockade disrupts pro-survival signaling pathways. Notably, this dual-targeted strategy retains synergistic efficacy even in p53-mutated contexts. This dual-targeted strategy may not only counteract resistance associated with monotherapy but also reduce dose-dependent toxicities through pharmacodynamic synergy. Importantly, leveraging the established clinical accessibility and safety profiles of both agents provides a pragmatic pathway to rapidly translate this combination into practice, offering a promising therapeutic advance for p53-mutated DLBCL patients with unmet needs.

Previous studies have established that p53 mutations are closely associated with dysregulation of the autophagy pathway [13]. Importantly, p53 mutations are frequently accompanied by increased NF-κB activity and decreased IκBα stability [9], while NF-κB itself can induce autophagic processes [15]. RNA sequencing analysis of p53-mutant versus wild-type DLBCL clinical specimens demonstrated a significant upregulation of autophagy in mutant cases. In DLBCL, heightened autophagic levels were associated with poorer disease-free survival (DFS) (Fig. 3J). In p53-positive DLBCL cell lines, the combination of chidamide and duvelisib significantly inhibited autophagic flux, with pathway enrichment analysis confirming significant enrichment of differentially expressed genes in autophagy-related pathways. These findings suggest that autophagy suppression through combination therapy may represent a potential mechanism for improving outcomes in p53-mutant DLBCL patients. In this study, we implemented transmission electron microscopy (TEM), immunofluorescence, and immunoblotting to validate that duvelisib and chidamide inhibit autophagy in p53-mutant DLBCL. Furthermore, both in vitro and in vivo experiments confirmed the therapeutic efficacy of this combination regimen, demonstrating that autophagy inhibition promotes apoptosis - a finding consistent with clinical specimen sequencing results.

NF-κB activation plays a critical role in maintaining tumor cell survival. The transcriptional and functional interplay between anti-apoptotic NF-κB and pro-apoptotic p53 pathways collectively determine tumor cell fate. Notably, the p65 subunit of NF-κB and p53 exhibit mutually antagonistic effects in regulating cellular proliferation, metabolism, and apoptosis. Following p53 mutation, aberrant activation of p65 may lead to predominant pro-survival signaling, thereby contributing to therapeutic resistance [11, 38]. In this study, we systematically evaluated the synergistic effects of chidamide and duvelisib across multiple experimental models, including in vitro cell lines, CDX models, and primary clinical samples. The combination therapy demonstrated potent inhibition of nuclear translocation of the NF-κB p65 subunit while concurrently enhancing apoptosis. Emerging evidence demonstrates that NF-κB promotes cytoprotective autophagy through upregulation of autophagy-related genes (e.g., BECN1 and LC3), while simultaneously suppressing apoptotic signaling via caspase inhibition, ultimately enhancing tumor cell survival under stress conditions. Consistent with this concept, RNA sequencing analysis of clinical specimens revealed significant enrichment of NF-κB signaling pathways in p53-mutant DLBCL compared to wild-type counterparts. Mechanistic investigations confirmed that this dual-drug regimen synergistically induces apoptosis in p53-mutant DLBCL cells through NF-κB pathway inhibition-mediated suppression of autophagy.

To elucidate the mechanism by which duvelisib interferes with NF-κB signaling, we examined its effects on p65 nuclear translocation. Our data demonstrated that duvelisib inhibits the phosphorylation of both IKK and IκBα primarily through selective inhibition of PI3Kδ. This results in increased stability of IκBα and a marked reduction in p65 nuclear translocation. To our knowledge, this regulatory mechanism of NF-κB signaling by duvelisib has not been previously reported. Intriguingly, chidamide exhibited unexpected NF-κB regulatory capacity. Mass spectrometry-based interactome analysis uncovered histone H1.5 as a novel IκBα-binding partner. Chidamide-induced HDAC2 inhibition enhanced H1.5 acetylation, which strengthened H1.5-IκBα complex stability and consequently delayed IκBα degradation. This epigenetic-pharmacological synergy resulted in sustained cytoplasmic retention of p65 and suppression of downstream survival signals. Moreover, we further demonstrated that chidamide specifically modulates acetylation at lysine 67 (K67) and lysine 93 (K93) residues of histone H1.5, thereby regulating its acetylation status. This investigation provides deeper insights into the molecular mechanism underlying the synergistic effects of the dual-drug combination therapy.

Current direct therapeutic strategies targeting mut-p53 (e.g., CDB3 synthetic peptides to restore mut-p53 DNA binding capacity or mut-p53 degradation) face multidimensional clinical challenges. The high heterogeneity of p53 mutations (missense/nonsense/deletions) imposes inherent complexity in developing broad-spectrum reactivators or degraders. Concurrently, restoring the structural and functional integrity of wild-type p53 within the tumor microenvironment encounters significant biological barriers. Interventions like CDB3-derived peptides are further constrained by pharmacokinetic limitations—including poor stability, inefficient delivery, and inadequate bioavailability—resulting in a scarcity of clinical-stage candidates. Existing regimens demonstrate only modest or variable efficacy with no significant survival improvement [39–41]. While decitabine-R-CHOP demonstrates modest survival improvement in p53-mutant DLBCL (3-year OS: 79.1% vs 89.5% in wild-type), substantial efficacy gaps persist with ongoing relapse risks [7, 42–45]. Our data reveal that the combination of duvelisib and chidamide achieves synergistic efficacy in p53-mutated DLBCL across patient-derived samples, cell lines, and CDX models, with activity approaching p53-WT models. This suggests potential to narrow the critical efficacy disparity between p53-mutant and WT disease, which is an unmet need inadequately addressed by current regimens. However, future studies comparing this combination clinically against Decitabine-R-CHOP are warranted. Notably, although duvelisib monotherapy exhibited mild immunotoxicities, the combination regimen demonstrated a favorable safety profile in xenograft models. This may be attributed to the suppression of IL-6 and IL-17 production by the HDAC inhibitor chidamide [46]. Nevertheless, further clinical toxicity monitoring remains warranted. In conclusion, this study provides compelling preclinical evidence that chidamide-duvelisib combination stabilizes IκBα through dual mechanisms (PI3Kδ inhibition and HDAC2-mediated histone H1.5 acetylation), suppressing NF-κB nuclear translocation and pro-survival autophagy in p53^+^ DLBCL (Supplementary Fig. 12). This innovative approach addresses critical therapeutic challenges in this high-risk population. While clinical validation is required, our findings delineate a therapeutic paradigm that preserves treatment efficacy in TP53-mutated DLBCL, potentially improving outcomes for patients with this aggressive lymphoma subtype.

## Methods

### Cell lines and reagents

The human p53^+^ DLBCL cell lines TMD8, Toledo, DB, and p53 wild-type (WT) DLBCL cell lines LR, MCA, WILL2 were provided by the Department of Hematology, The First Affiliated Hospital of Xiamen University (Fujian, China). All cell lines were tested and identified. 10% fetal bovine serum (FBS, Excell Bio, Shanghai, China), 100 U/ml penicillin, and 100 μg/ml streptomycin (Invitrogen, MA, USA) were added to RPMI-1640 medium (Basal Media, Shanghai, China) for cell culture. All cell lines were maintained at 37 °C in a 5% CO_2_ incubator. Duvelisib (T1988) was purchased from TargetMol (MA, USA) and dissolved in dimethyl sulfoxide (DMSO) (Sigma, MO, USA) to obtain a stock solution at a concentration of 50 mM. Chidamide (CS055, >95% purity) was obtained from Chipscreen Bioscience Co., Ltd. (Shenzhen, Guangdong, China) and dissolved in DMSO to achieve a 50 mM stock solution. When administered in mice, the solvent formulation for duvelisib consisted of 10% DMSO, 40% PEG 300 (MedChemExpress, HY-Y0873, USA), 5% Tween 80 (MedChemExpress, HY-Y1891, USA), and 45% PBS (P1020, Solarbio, China). Chidamide was diluted with 0.2% (w/v) CMC-Na suspension and injected intraperitoneally. PI3kγ inhibitor AZ2 (HY-111570) and PI3K-in-50 (HY-160283) were purchased from MedChemExpress, USA. In cell-based experiments, DMSO served as the vehicle control. To ensure that DMSO exposure did not affect cell proliferation or viability, it was diluted in culture medium to achieve a final concentration of 0.05% (v/v).

### Cell viability assay

P53^+^ DLBCL cells (TMD8, Toledo, DB, 2 × 10^4^ cells per well) and p53-WT DLBCL cells (LR, MCA, WILL2) were plated in 100 μL of medium in 96-well plates and treated with designated doses of duvelisib or chidamide, either alone or in combination. The cells were incubated at 37 °C in a 5% CO_2_ incubator with 100% humidity for 24 and 48 h. Absorbance was then measured at 450 nm using a microplate reader (ELx800, BioTek Laboratories, Shoreline, WA, USA). The results are expressed as the percentage of viable cells relative to the control group, with three replicates per condition. Statistical analysis and graphing were performed using GraphPad Prism 8.

### Primary samples

This study was approved by the Ethics Committee of the First Affiliated Hospital of Xiamen University and conducted in accordance with the principles of the Declaration of Helsinki. All patients provided written informed consent. Clinical specimens from 6 cases of p53-mutated DLBCL and 6 cases of p53 wild-type DLBCL were collected. An appropriate amount of lymph node tissue was excised, mechanically ground, and processed for cell isolation. After extraction, cells were centrifuged to remove the supernatant. Red blood cells were lysed using erythrocyte lysis buffer (Solarbio, R1010, China).

### Analysis of apoptosis

Apoptosis was assessed by treating cells with duvelisib, chidamide, or their combination for 24 and 48 h (primary cells were treated for 24 h), followed by staining with Annexin-V-APC/PI (BD Pharmingen, USA) for 15 min at room temperature in the dark, according to the manufacturer’s protocol. Cells were analyzed using a NovoCyte flow cytometer, and data were processed with NovoExpress software (ACEA Biosciences, California, USA) to determine the percentage of Annexin-V positive cells.

### RNA sequencing

DB cells were treated with chidamide (10 µM) and duvelisib (0.8 µM), either alone or in combination, for 24 h. Primary DLBCL cells isolated from clinical specimens (*n* = 12; 6 with p53 mutations and 6 wild-type) were processed using the described protocols above. Total RNA was extracted from cells, and after quality assessment, RNA sequencing (RNA-seq) was performed using the Illumina HiSeq 2500 platform. Briefly, the RNA samples were fragmented into short pieces, which were then used as templates for double-stranded cDNA synthesis. The synthesized cDNA was purified, enriched via polymerase chain reaction (PCR) amplification, and the resulting library was sequenced.

### Western blotting

Whole-cell lysates were prepared and quantified from each sample. After electrophoresis and transfer to a PVDF membrane, the proteins were incubated with the following primary antibodies, followed by HRP-conjugated secondary antibodies (1:10,000, Abcam, UK). The following antibodies were used in this research: anti-PARP (#9532, 1:1000, Cell Signaling Technology, MA, USA), anti-cleaved PARP (#9544, CST), anti-c-myc (#5605, CST), anti-caspase9 (#9502, CST), anti-cleaved-caspase9 (#20750, CST), anti-caspase3 (#9662, CST), anti-cleaved-caspase3 (#9661, CST), anti-beclin1 (3738S, CST), anti-Akt (#9272, CST), anti-p-Akt (Ser473)(#4060, CST), anti-HDAC1 (83624-1, proteintech), anti-HDAC2 (12922-3, proteintech), anti-HDAC3 (#3949, CST), anti-HDAC10 (ab108934, 1:1000, abcam), anti-GAPDH (#5174, CST), anti-NF-κB-p65 (#8242, CST), anti-p-NF-κB-p65 (Ser536)(#3033, CST), anti-Acetylated-Lysine (#9441, CST), anti-Acetylated-Lysine (#9681, CST), anti-IκBα (#4814, CST), anti-IκBα (#4812, 1:1000, CST), anti-p-IκBα (Ser32)(#BM4411, 1:1000, boster), anti-IKKα/β (#ET1611-23, 1:1000, huabio), anti-p-IKKα/β (Ser176/180)(#2697, 1:1000, CST) and anti-Histone H1.5 (ab18208, abcam). Protein signals were then detected using the ECL Western Blotting Detection Kit (GeneFlow, Staffordshire, UK). The intensity of each band was determined using ImageJ (RRID: SCR_003070).

### P53^+^ DLBCL xenograft in mice

The 6-week-old female CB17/Icr-Prkdcscid/IcrlcoCrl (CB-17 SCID) mice used in this study were purchased from Xiamen University Animal Care and maintained in a pathogen-free environment. Following 1 Gly irradiation, 1×10⁷ TMD8 cells were subcutaneously inoculated into mice. Tumor growth was monitored, and when tumor volumes reached 50–100 mm³, 32 mice were randomly allocated into four groups (8 mice per group; 4 for survival analysis and 4 for specimen collection): (1) PBS control, (2) duvelisib monotherapy (10 mg/kg/day), (3) chidamide monotherapy (10 mg/kg/day) and (4) duvelisib and chidamide combination therapy (duvelisib 10 mg/kg/day and chidamide 10 mg/kg/day). The drug dose was determined based on previous studies and preliminary experiments [47–49]. Tumor size and body weight were measured daily throughout the 14-day treatment period. After 14 days, 4 mice per group were euthanized following orbital blood sampling, and liver, kidney, and tumor tissues were collected for subsequent analysis. Tumor volumes were measured and weighed for subsequent histological examinations, including H&E staining, immunohistochemistry, and TUNEL staining. Tumor volume was calculated using the formula: V = 1/2 (L × W²), where L is the length and W is the width of the tumor. Survival times were recorded for the remaining mice. Mice were euthanized when their tumor volume reached 1500 mm³, and survival curves were plotted. Following a double-blind protocol, the study was approved by the Ethics Committee of Xiamen University and complies with the Declaration of Helsinki.

### Small interfering RNA (siRNA) and plasmids, transient transfection

DB cells were transfected with siRNA targeting HDAC1 (SC131972; Miaoling, China), HDAC2 (SC131974; Miaoling, China), HDAC3 (SC131971; Miaoling, China), HDAC10 (SC131968; Miaoling, China) or plasmids encoding Flag-Histone H1.5 and its mutants (K37 [G65127], K67 [G65128], K49 [G65129], K88 [G65130], K93 [G65131]; Miaoling, China) using Lipofectamine™ 3000 Transfection Reagent (Invitrogen; L3000015), according to the manufacturer’s instructions.

### Establishment of stable cell lines

To establish stable RELA/BECN1-overexpressing cell lines, the coding sequences of RELA and BECN1 were cloned into the lentiviral expression vector pLV6ltr-ZsGreen-puro-CMV. Following sequencing verification of the recombinant plasmids pLV6ltr-ZsGreen-puro-CMV-RELA and pLV6ltr-ZsGreen-puro-CMV-BECN1, they were co-transfected with packaging plasmids (psPAX2 and pMD2.G) into HEK293T cells via Lipo293™ Transfection Reagent (Beyotime, C0521). Viral supernatants were harvested 72 h post-transfection and subsequently used to infect DB cells in the presence of 8 μg/mL Polybrene (Sigma-Aldrich) to enhance transduction efficiency. After 24 h incubation, the medium was replaced with fresh complete medium, followed by 2 μg/mL puromycin selection for 7–10 days to establish stable polyclonal populations. Successful overexpression was validated by quantitative real-time polymerase chain reaction (qRT‒PCR) and western blotting.

### RNA isolation, reverse transcription, and qRT‒PCR

Total RNA isolation from DB-RELA-overexpressing and DB-BECN1-overexpressing cell cultures was performed utilizing TRIzol reagent (Invitrogen, 155960266, USA). Subsequent cDNA synthesis was carried out with the NovoScript Plus All-In-One 1st Strand cDNA Synthesis SuperMix Kit (Novoprotein, E047-01A, China) following standardized reverse transcription protocols. Quantitative real-time PCR analysis was conducted using SYBR qPCR SuperMix (Novoprotein, E096-01A, China) under optimized amplification conditions: initial denaturation at 95 °C for 60 s, followed by 40 cycles of denaturation (95 °C, 20 s) and annealing/extension (60 °C, 60 s). Amplification signals were monitored in real-time using the ABI 7900HT PCR detection system (QuantStudio6 Flex, Thermo Fisher, USA), with GAPDH serving as the endogenous reference gene for data normalization. All reactions were performed in technical triplicates with appropriate negative controls. Primer sequences for target genes are provided in Supplementary Table 1.

### Histological analysis

Tissues from liver, kidney, and tumor specimens of xenograft mice were fixed in formalin, embedded in paraffin, and sectioned. After deparaffinization with xylene, one set of sections was stained with hematoxylin and eosin (H&E). The remaining sections were subjected to manual immunohistochemical staining using the following primary antibodies: Ki67 (1:800, #9449, CST) and PCNA (1:4000, #13110, CST). Subsequently, additional tumor sections were stained using immunofluorescence according to the manufacturer’s instructions (C1086, Beyotime). Analysis was performed using a fluorescence microscope (Olympus VS200, Japan). The staining signals were scored based on the percentage of positive tumor cells. Image analysis was carried out with FV31S SW software (Fukuda Denshi Co., Ltd., Japan).

### Enzyme-Linked Immunosorbent Assay (ELISA)

The serum of mice was collected, and the cytokines IL-6 and IL-17 were measured using ELISA kits (Mlbio, Shanghai, China) according to the manufacturer’s instructions. The absorption at 450 nm was measured by a microplate reader (ELx800, BioTek Laboratories, Shoreline, WA, USA).

### Transmission electron microscopy analysis

Cells were seeded in 10 cm culture dishes and treated with duvelisib, chidamide, their combination, or left untreated. After 24 h, the culture medium was removed by centrifugation, and the cells were fixed with electron microscopy fixative at room temperature for 30 min, protected from light, and then stored at 4 °C. Electron micrographs were captured by Servicebio Technology (Wuhan, China).

### Immunoprecipitation assay

Cells were lysed with RIPA buffer (Beyotime, P0013) containing phosphatase inhibitors (APExBIO, K1007, USA) and protease inhibitors (APExBIO, K1015, USA) while gently shaking at 4 °C for 1 h. The supernatant was then incubated overnight at 4 °C with the specified antibody and magnetic beads, followed by five washes with IP buffer. The immunoprecipitated complexes were boiled in sample buffer at 100 °C for 10 min. The IgG group served as the negative control, which consisted of lysate supernatant from untreated cells combined with species-matched nonspecific IgG antibody. Proteins were analyzed by western blotting.

### Immunofluorescence staining

Cells were seeded onto poly-L-lysine-coated coverslips (Labselect, 18122). After the specified treatments, the cells were fixed with 4% paraformaldehyde (Solarbio, P1110) and permeabilized with 0.2% Triton X-100 (P0096, Beyotime). Following incubation with primary and secondary antibodies (Elab Fluor® 488 conjugated, E-AB-1055; Elab Fluor® 594 conjugated, E-AB-1060), fluorescence intensity was observed using a FLUOVIEW FV3000 confocal microscope (Evident, Japan). Representative images of cells were captured for analysis.

### Mass spectrometry (MS) analysis

In this study, we employed mass spectrometry (MS) analysis to achieve two main objectives. First, to identify proteins interacting with IκBα, DB cells were treated with chidamide or left untreated for 24 h. Immunoprecipitation was performed using IκBα antibodies, following the method described in the manuscript. After sample preparation, the proteins were separated by SDS-PAGE. The corresponding bands of IκBα were excised and subjected to trypsin digestion. The resulting peptides were analyzed using LC-MS (Thermo Fisher Easy-nLC 1000 coupled with Thermo Fisher LTQ Orbitrap ETD). The data were processed using MASCOT software to construct the protein-protein interaction network of the proteins identified by LC-MS.

Secondly, to analyze the acetylation sites of proteins interacting with IκBα, peptides were analyzed using a mass spectrometry system consisting of a Dionex Ultimate 3000 RSLC nano LC system (Thermo Fisher, Waltham, MA, USA) coupled with a Q-Exactive mass spectrometer (Thermo Fisher, Waltham, MA, USA). Data were searched against the UniProt human database using an internal Mascot server (version 2.4.1, Matrix Science, Boston, MA, USA). Acetylated peptides were manually verified by inspecting the MS/MS spectra to pinpoint the precise acetylation sites. All analyses were conducted by Jiyun Biotech (Shanghai, China).

### Statistical analysis

Based on the principle of replication, all biological and technical replicates were performed more than three times. Statistical analysis was performed using GraphPad Prism 8.0. Continuous variables are expressed as the mean ± standard deviation (SD). Differences between two independent groups were assessed using the independent-sample *t*-test. For multiple group comparisons, one-way analysis of variance (ANOVA) was applied, with *p < 0.05, **p < 0.01, ***p < 0.001, and ****p < 0.0001. Homogeneity of variance was confirmed when performing statistical comparisons between groups.

## Supplementary information

Supplementary table 1-2

Supplementary figure 1-12

original wblots

## Data Availability

The datasets presented in this study are available from the corresponding authors with reasonable requests.
