# Supplementary material for: Combination of HDAC inhibitor and PI3K inhibitor suppresses autophagy and induces apoptosis via cytoplasmic IκBα stabilization in p53-mutant diffuse large B-cell lymphoma
Source: Cell Death Discov. 2025 Oct 6;11:445. doi: 10.1038/s41420-025-02756-7 (PMC12501026; doi:10.1038/s41420-025-02756-7)
Supplement: Supplementary file 1 — Supplementary table 1-2 [file 41420_2025_2756_MOESM1_ESM.docx]

**Supplementary table 1** Primer Sequences for qPCR Validation of Expression Levels in RELA- and BECN1-Overexpressing Cell Lines

| BECN1 | | RELA | |
| --- | --- | --- | --- |
| BECN1-qF | GCAGCCAGCCCCTGAAAC | RELA-qF | CGAGCTTGTAGGAAAGGACTG |
| BECN1-qR | CTCCTCTCCTGAGTTAGTCTCTTCC | RELA-qR | GGGTTGTTGTTGGTCTGGATG |
| GAPDH-qF | CGTCATGGGTGTGAACCATG | GAPDH-qF | CGTGTCCGTTGTGGATCTGA |
| GAPDH-qR | GGACTGTGGTCATGAGTCCT | GAPDH-qR | TCACTGTTGAAGTCGCAGGAG |

**Supplementary table 2** Clinical data of DLBCL patients for RNA-seq sequencing analysis

| **Patient No.** | **Gender** | **Age** | **clinical**  **stages** | **WBC**  **( × 10^9^ /L)** | **HGB**  **(g/L)** | **PLT**  **(*10^9^/L)** | **Lymphocyte%** | **monocyte%** | **LDH**  **（U/L）** | **P53**  **Status** |
| --- | --- | --- | --- | --- | --- | --- | --- | --- | --- | --- |
| 1 | Male | 46 | IIIB | 4.27 | 135 | 250 | 34.7 | 21.1 | 172 | Mutant |
| 2 | Female | 64 | IVA | 21.88 | 118 | 87 | 17.5 | 17.8 | 2740 | Mutant |
| 3 | Male | 53 | IIA | 6.44 | 135 | 258 | 8.9 | 3.4 | 188 | Mutant |
| 4 | Female | 56 | IVA | 6.06 | 136 | 205 | 25.4 | 4.3 | 139 | Mutant |
| 5 | Male | 72 | IIA | 5.49 | 127 | 210 | 26.8 | 9.1 | 182 | Mutant |
| 6 | Male | 46 | IVA | 4.26 | 137 | 156 | 19.7 | 13.4 | 190 | Mutant |
| 7 | Male | 82 | IVB | 14.79 | 81 | 51 | 8.2 | 8.7 | 315 | Wild-type |
| 8 | Male | 81 | IVB | 6.8 | 125 | 208 | 14.7 | 11.2 | 1419 | Wild-type |
| 9 | Female | 69 | IVA | 6.43 | 113 | 345 | 20.2 | 11.5 | 295 | Wild-type |
| 10 | Female | 75 | IVB | 12.51 | 109 | 364 | 15.7 | 8 | 258 | Wild-type |
| 11 | Male | 55 | IVA | 7.65 | 108 | 356 | 23.9 | 15.9 | 166 | Wild-type |
| 12 | Female | 56 | IIIB | 5.55 | 116 | 298 | 30.2 | 18.88 | 179 | Wild-type |
