# Supplementary material for: Combination of HDAC inhibitor and PI3K inhibitor suppresses autophagy and induces apoptosis via cytoplasmic IκBα stabilization in p53-mutant diffuse large B-cell lymphoma
Source: Cell Death Discov. 2025 Oct 6;11:445. doi: 10.1038/s41420-025-02756-7 (PMC12501026; doi:10.1038/s41420-025-02756-7)
Supplement: Supplementary file 2 — Supplementary figure 1-12 [file 41420_2025_2756_MOESM2_ESM.pdf]

Supplementary Figure 1

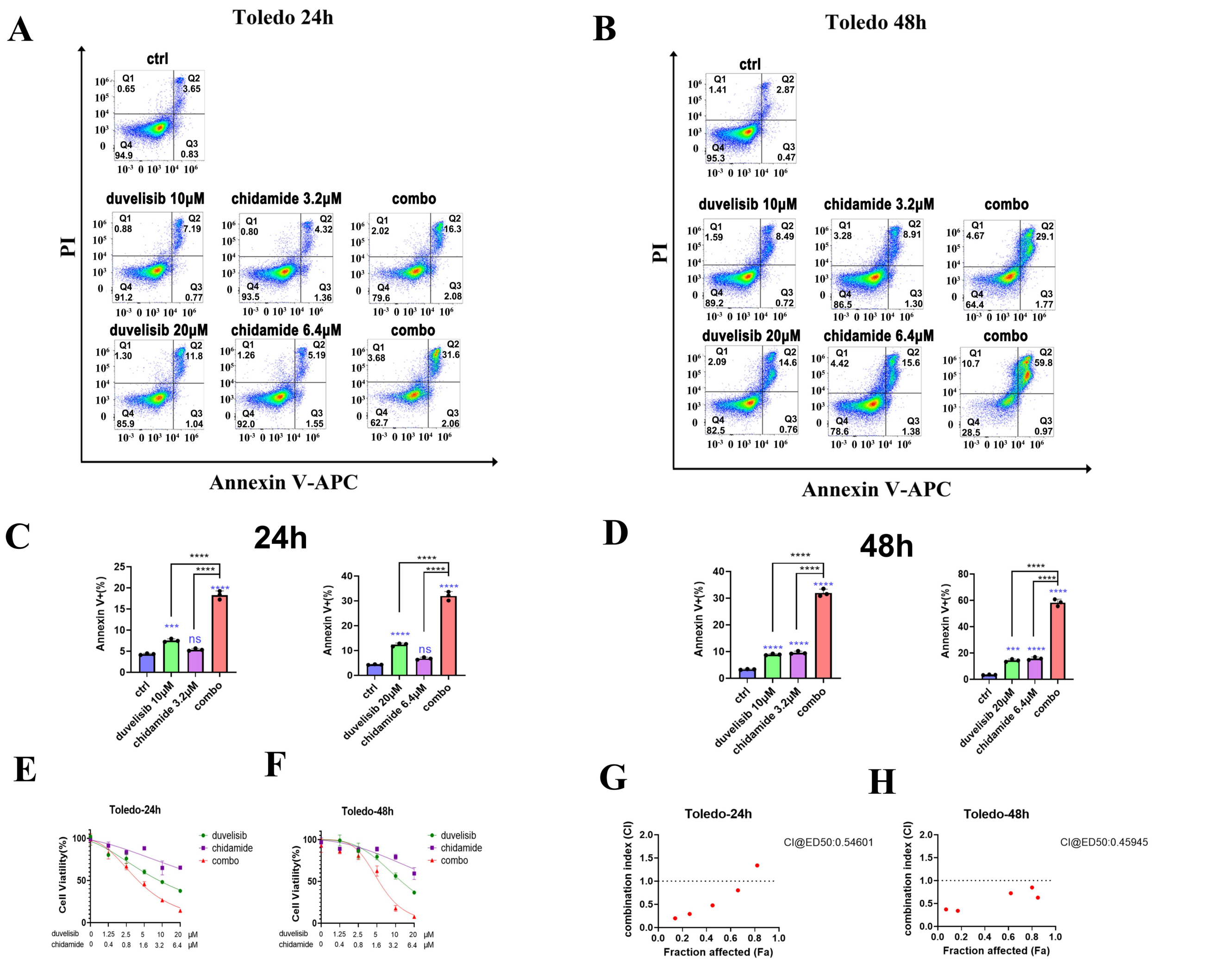

Supplementary Figure 1. Combined Treatment with Chidamide and Duvelisib Significantly Enhances Apoptosis and Inhibits Proliferation in Toledo Cells

(A-D) Toledo cells were exposed to chidamide, duvelisib, or their combination at specified concentrations for 24 or 48 hours. Apoptosis levels were measured using Annexin V/PI dual staining flow cytometry, with apoptotic populations defined as Annexin V-positive cells. Results are presented as mean ± SD from three independent experiments. Statistical analysis was performed using one-way ANOVA. **(E-H)** Following treatment with designated concentrations of chidamide and duvelisib for 24 or 48 hours, respectively, the suppressive effects on cellular proliferation were evaluated using a CCK-8 assay kit. Drug interaction patterns (synergism, additive effect, or antagonism) were systematically evaluated through combination index (CI) calculations using CompuSyn software.

Supplementary Figure 2

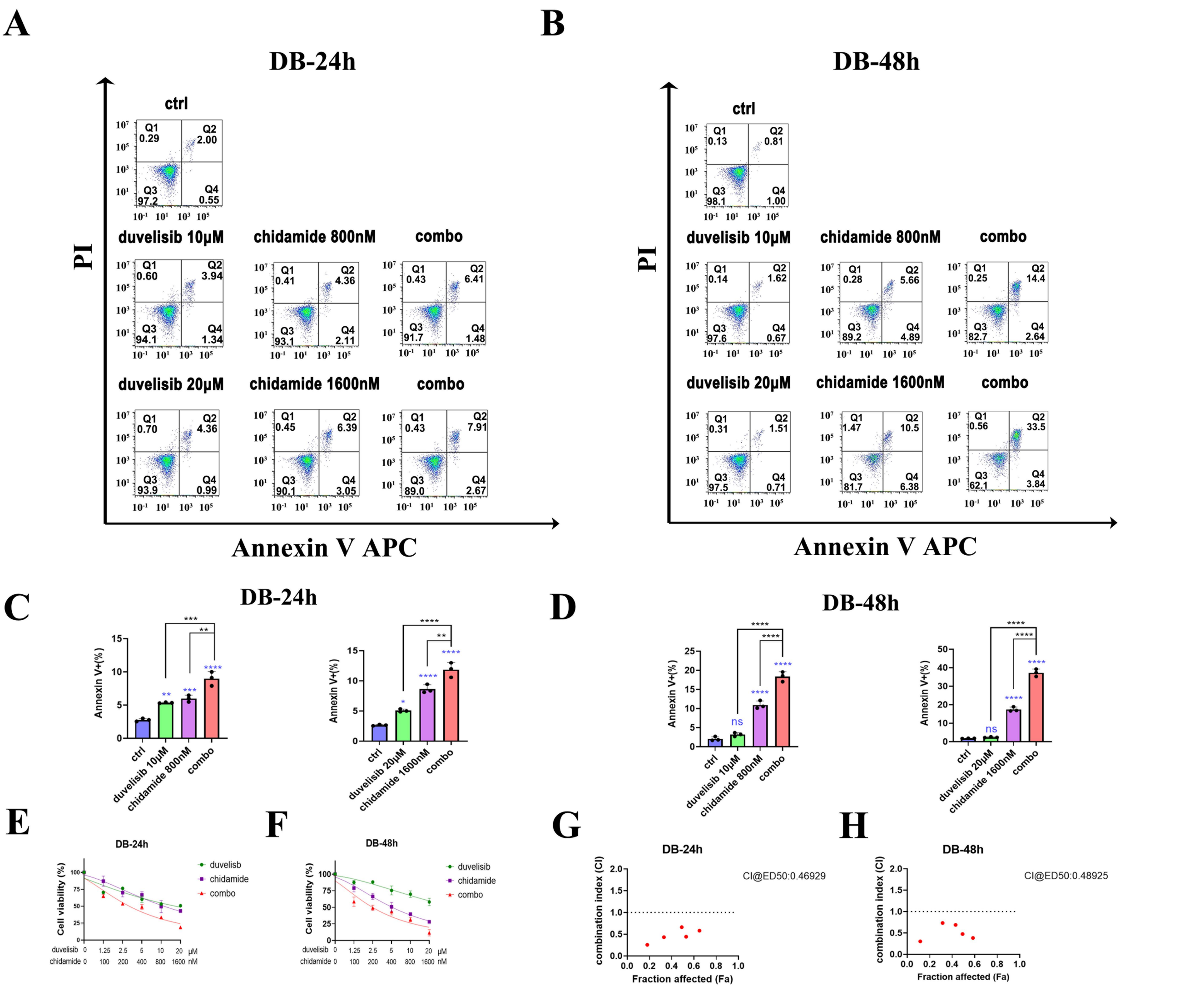

Supplementary Figure 2. Combined Treatment with Chidamide and Duvelisib Significantly Enhances Apoptosis and Inhibits Proliferation in DB Cells

(A-**D)** DB cells were incubated with chidamide, duvelisib, or combined agents at specified doses for 24/48 hours. Apoptotic cell death was assessed using Annexin V/PI dual staining followed by flow cytometry (Annexin V^+ populations considered apoptotic). Triplicate experimental data are shown as mean ± SD. Significance thresholds were established through one-way ANOVA. **(E-H)** Following 24 or 48-hour exposure to chidamide and duvelisib at prescribed concentrations, cellular proliferation suppression was measured via CCK-8 assay. Synergistic potential was mathematically modeled using CompuSyn's combination index (CI) algorithm, applying the Chou-Talalay principle for drug interaction characterization.

Supplementary Figure 3

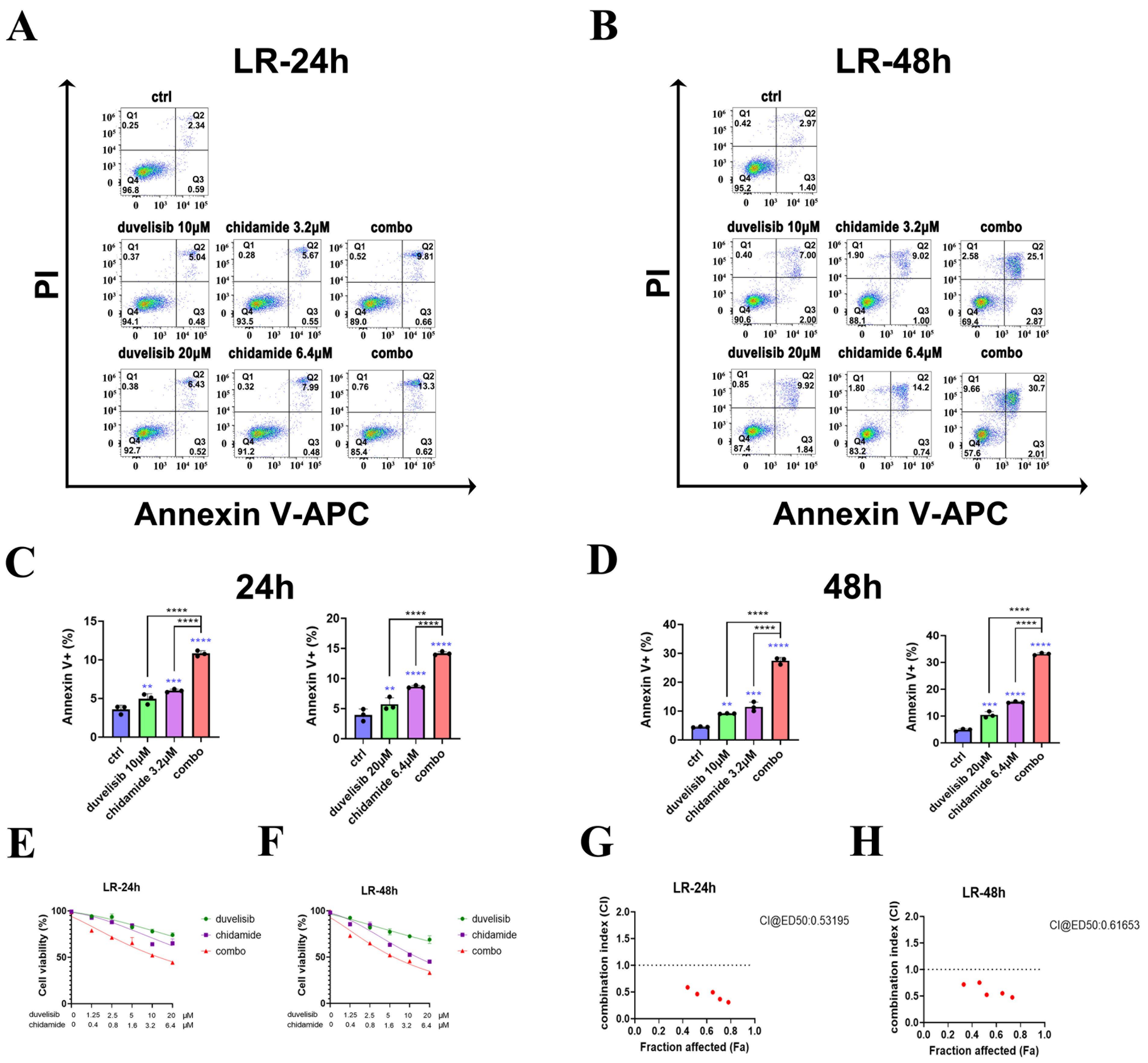

Supplementary Figure 3. The therapeutic efficacy of combined Chidamide and Duvelisib treatment in p53-WT LR cells.

(A-D) LR cells were treated with chidamide, duvelisib, or their combination for 24/48 hours. Apoptosis was measured by Annexin V/PI staining and flow cytometry. Data (mean ± SD, n=3) were analyzed by one-way ANOVA. **(E-H)** Proliferation inhibition after 24/48-hour drug exposure was assessed by CCK-8 assay. Synergy was calculated using CompuSyn's combination index (CI) based on the Chou-Talalay principle.

Supplementary Figure 4

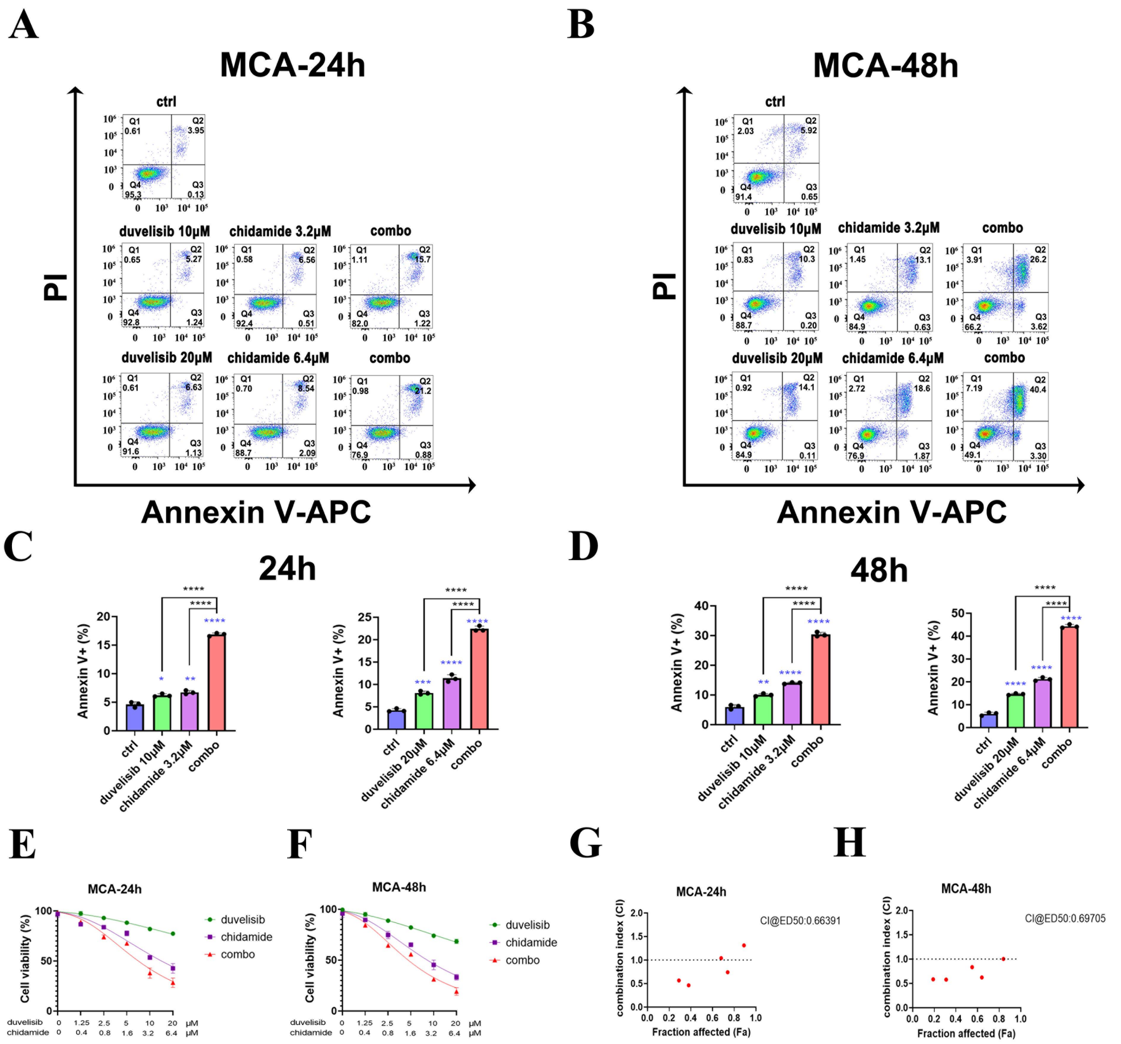

Supplementary Figure 4. Synergistic effects of Chidamide and Duvelisib co-treatment in p53-WT MCA cells.

(A-D) MCA cells were treated with chidamide, duvelisib, or their combination for 24 or 48 hours. Apoptotic cells (Annexin V+) were detected by flow cytometry. Data represent mean ± SD of triplicate experiments by one-way ANOVA. **(E-H)** Cell proliferation was measured by CCK-8 assay after 24/48 h treatment. Drug synergy was analyzed using CompuSyn software (Chou-Talalay method, combination index CI).

Supplementary Figure 5

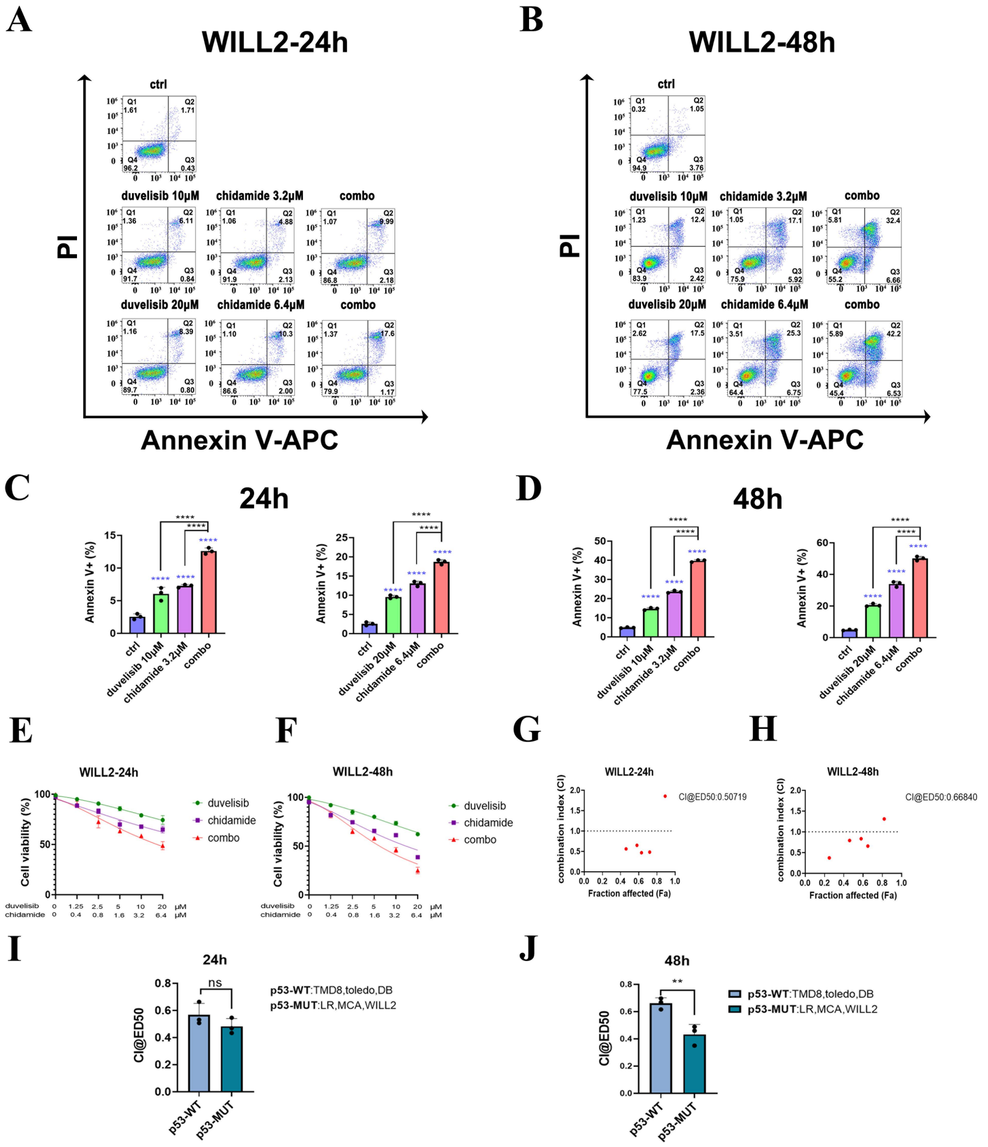

Supplementary Figure 5. Efficacy assessment of Chidamide and Duvelisib combination in p53-WT WILL2 cells. (A-D) WILL2 cells were treated with chidamide, duvelisib, or their combination for 24 or 48 hours. Apoptotic rates were assessed by Annexin V/PI staining followed by flow cytometry, with Annexin V-positive cells considered apoptotic. Data from three independent experiments are presented as mean ± standard deviation (SD). Statistical significance was analyzed using one-way ANOVA. **(E-H)** The anti-proliferative effects of the drugs were examined using the CCK-8 assay following 24 or 48 hours of treatment. To assess drug interaction, we calculated the combination index (CI) using CompuSyn software based on the Chou-Talalay principle. **(I-J)** The combined effects of duvelisib and chidamide were compared between three p53 wild-type DLBCL cell lines (LR, MCA, WILL2) and three p53-mutated DLBCL cell lines (TMD8, Toledo, DB).

Supplementary Figure 6

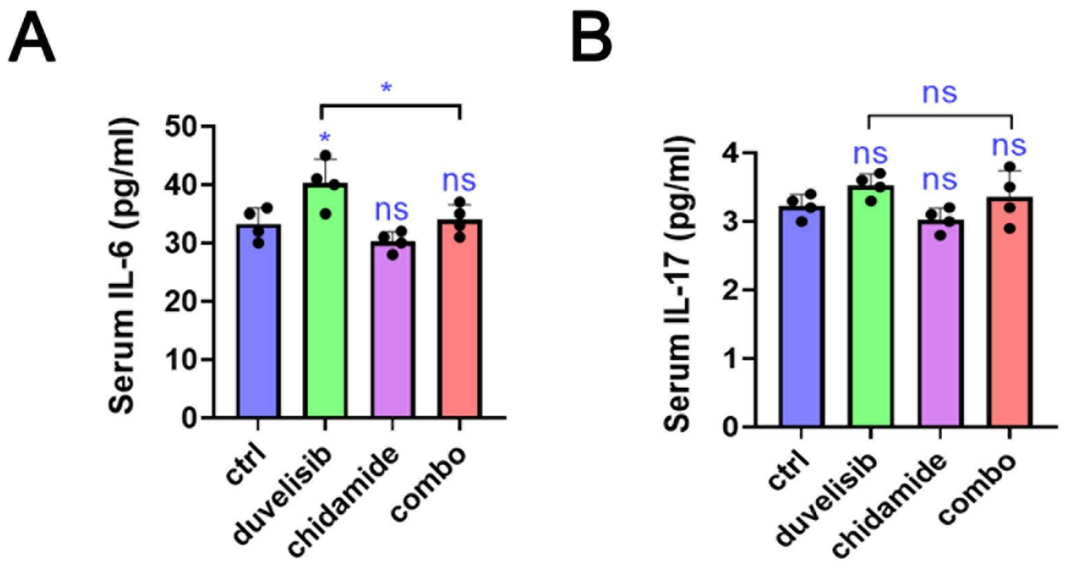

Supplementary Figure 6. Serum levels of IL-6 (A) and IL-17 (B) in mice were measured by ELISA

Supplementary Figure 7

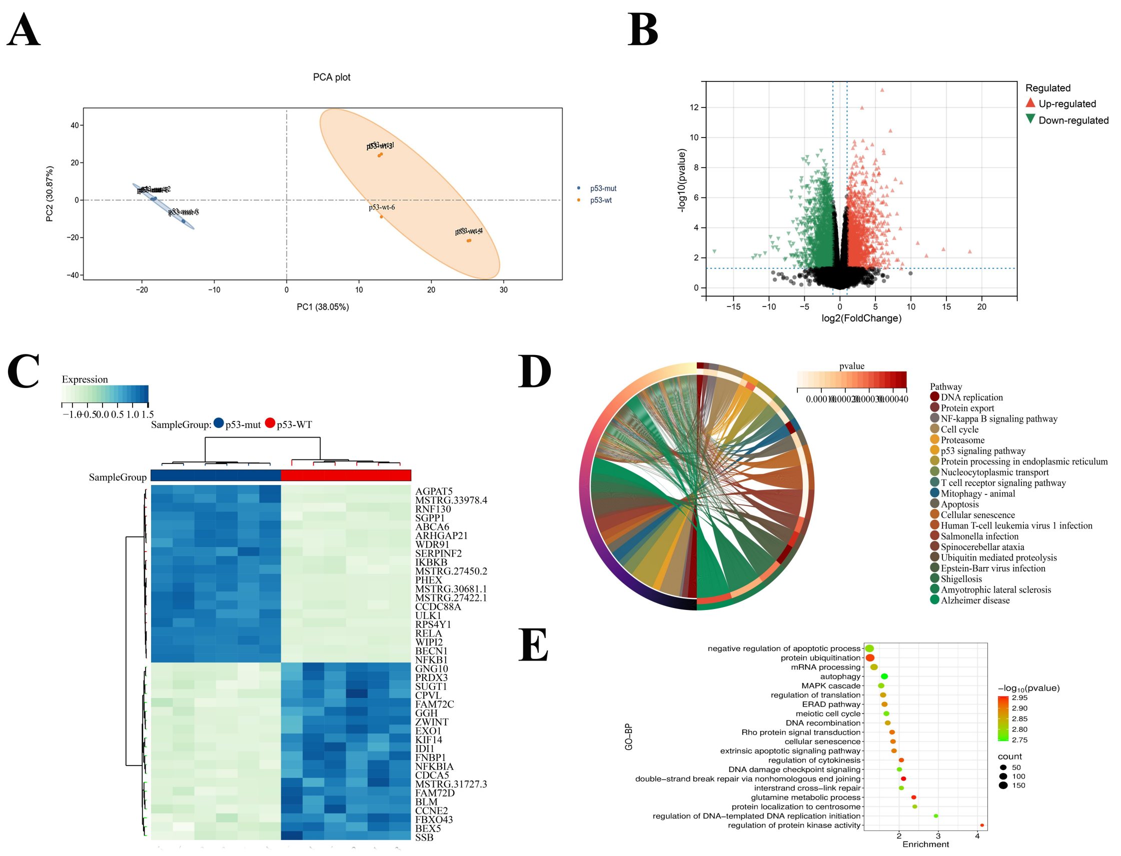

Supplementary Figure 7. Comparative analysis of differentially expressed genes and signaling pathway discrepancies between TP53-mutated and wild-type diffuse large B-cell lymphoma patients

Tumor specimens were collected from 6 cases of p53-mutant diffuse large B-cell lymphoma (DLBCL) and 6 cases of p53 wild-type DLBCL. RNA sequencing (RNA-seq) was performed to profile the transcriptomic landscape, followed by principal component analysis (PCA) **(A)** and differentially expressed genes (DEGs) analysis **(B-C)**. Subsequently, the identified DEGs were subjected to KEGG pathway enrichment analysis **(D)** and GO enrichment analysis **(E)** to elucidate their functional characteristics and biological pathway associations.

Supplementary Figure 8

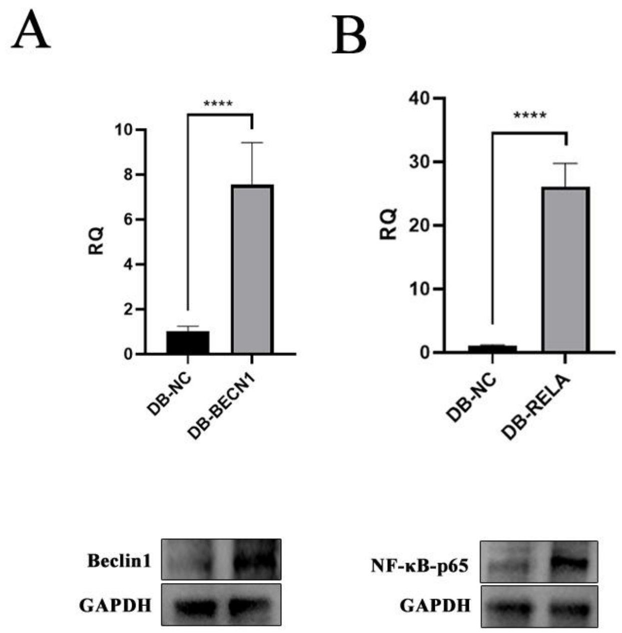

Supplementary Figure 8. Validation of RELA and BECN1 Expression Levels in Overexpressing Cell Lines Using qPCR and western-blot

(A) Validation of DB-BECN1 Overexpressing Cell Line Construction.

(B) Validation of DB-RELA Overexpressing Cell Line Construction.

Supplementary Figure 9

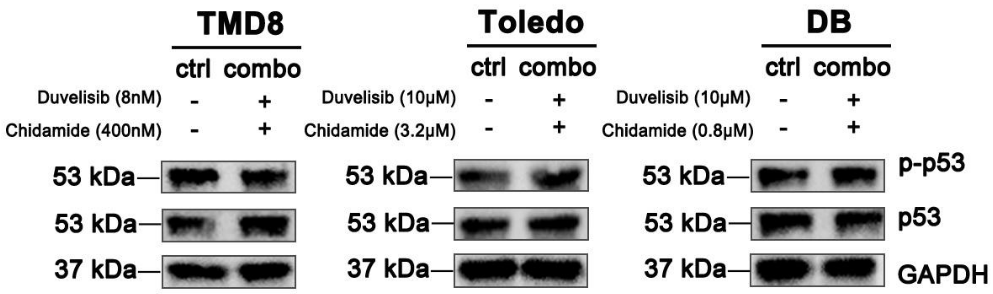

Supplementary Figure 9. Western-blot was performed to assess changes in p53 expression before and after combination therapy with chidamide and duvelisib

Supplementary Figure 10

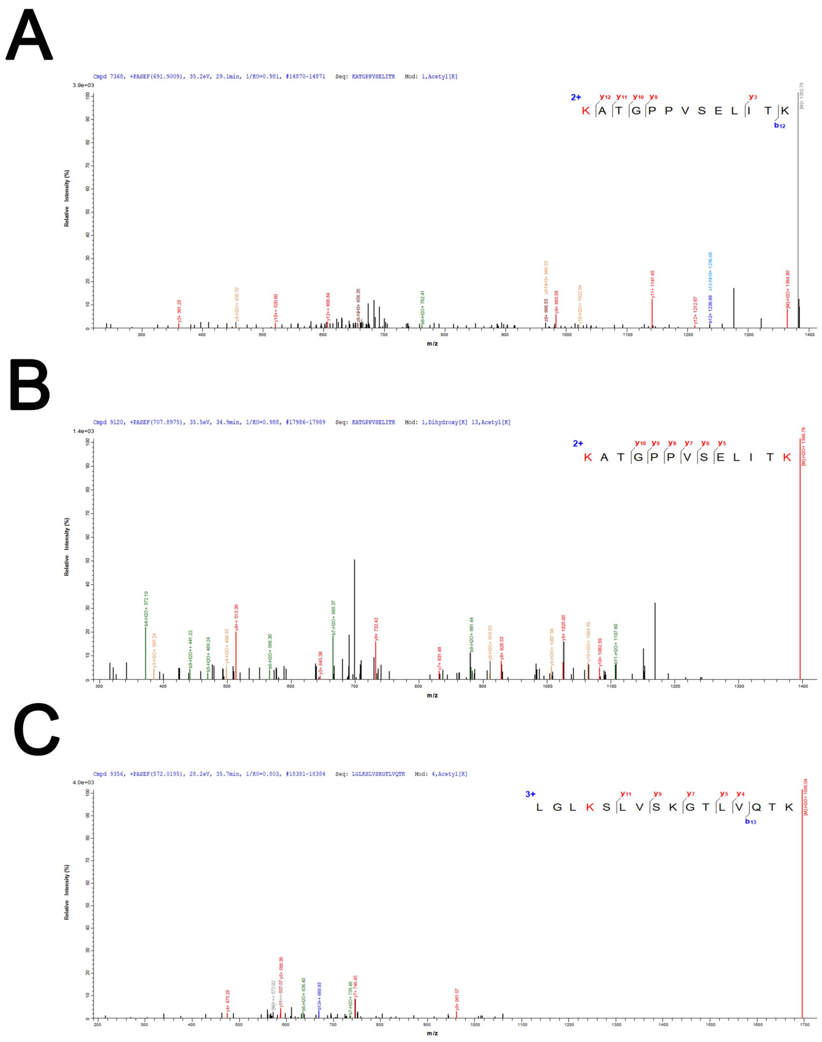

Supplementary Figure 10. Identification of acetylation modification site of histone H1.5 by mass spectrometry

Mass spectrometry identified five histone H1.5 acetylation sites with A-scores >13 after chidamide treatment. The three acetylated mutation sites K37 **(A)**, K49 **(B)**, and K88 **(C)** were found to not significantly change the acetylation status of histone H1.5.

Supplementary Figure 11

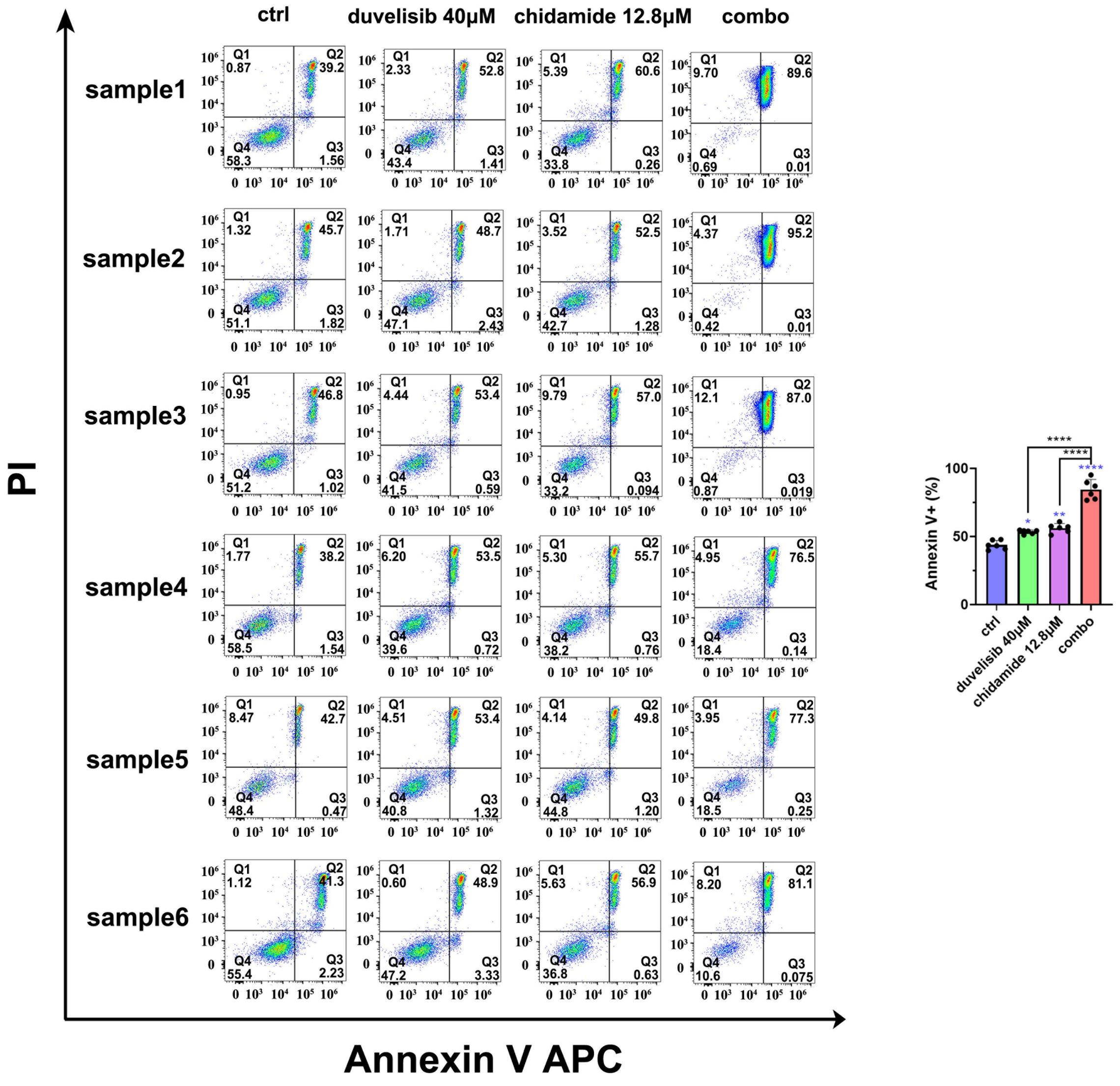

Supplementary Figure 11. The combined administration of chidamide and duvelisib demonstrates significant apoptotic induction in primary diffuse large B-cell lymphoma (DLBCL) specimens with wild-type p53.

Supplementary Figure 12

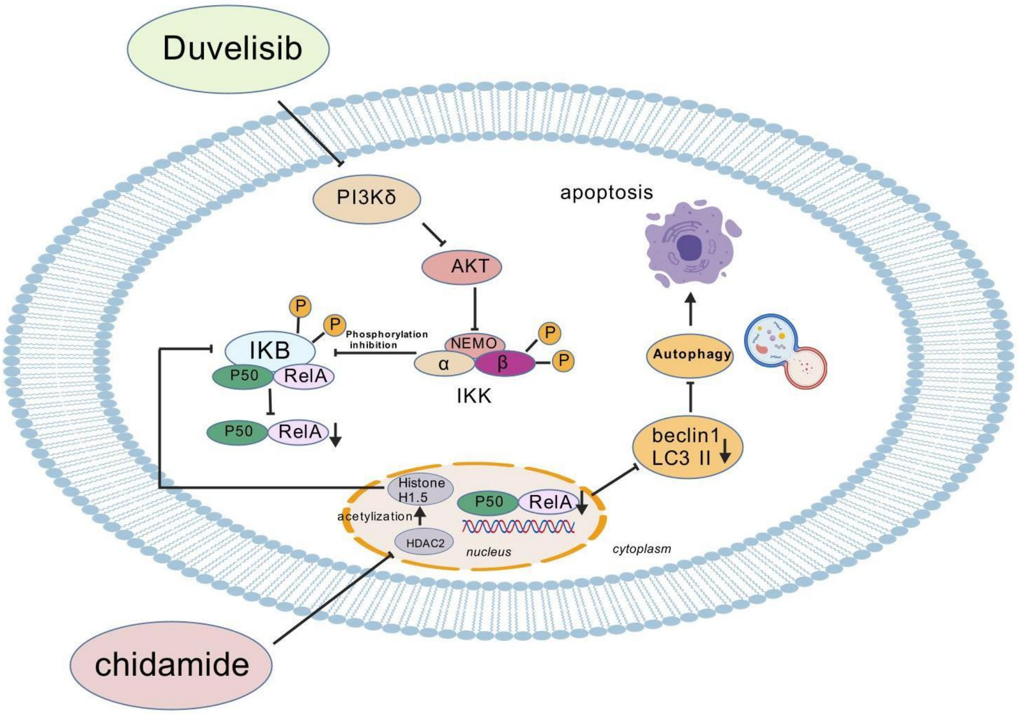

Supplementary Figure 12. Proposed Mechanism of This Study: The Duvelisib-Chidamide combination inhibits PI3Kδ and HDAC2, stabilizing cytoplasmic IκB to block NF-κB-p65 phosphorylation and nuclear translocation. This blockade subsequently suppresses autophagy, ultimately leading to tumor proliferation inhibition and apoptosis promotion.
