# Supplementary material for: Combination of HDAC inhibitor and PI3K inhibitor suppresses autophagy and induces apoptosis via cytoplasmic IκBα stabilization in p53-mutant diffuse large B-cell lymphoma
Source: Cell Death Discov. 2025 Oct 6;11:445. doi: 10.1038/s41420-025-02756-7 (PMC12501026; doi:10.1038/s41420-025-02756-7)
Supplement: Supplementary file 3 — original wblots [file 41420_2025_2756_MOESM3_ESM.pdf]

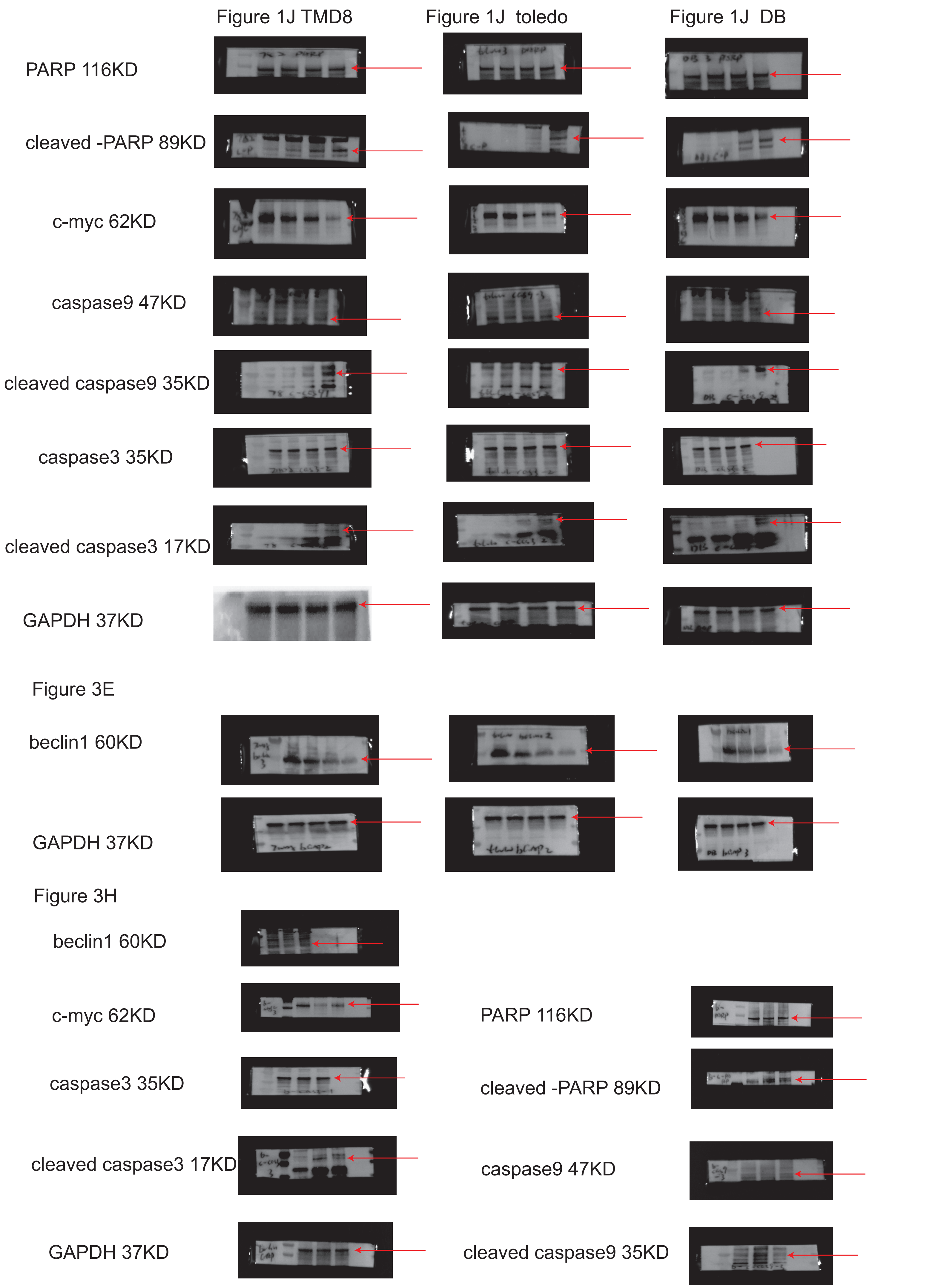

Figure 1J TMD8
Figure 1J toledo
Figure 1J DB
PARP 116KD
cleaved -PARP 89KD
c-myc 62KD
caspase9 47KD
cleaved caspase9 35KD
caspase3 35KD
cleaved caspase3 17KD
GAPDH 37KD
Figure 3E
beclin1 60KD
GAPDH 37KD
Figure 3H
beclin1 60KD
c-myc 62KD
caspase3 35KD
cleaved caspase3 17KD
GAPDH 37KD
PARP 116KD
cleaved -PARP 89KD
caspase9 47KD
cleaved caspase9 35KD

Figure 4D

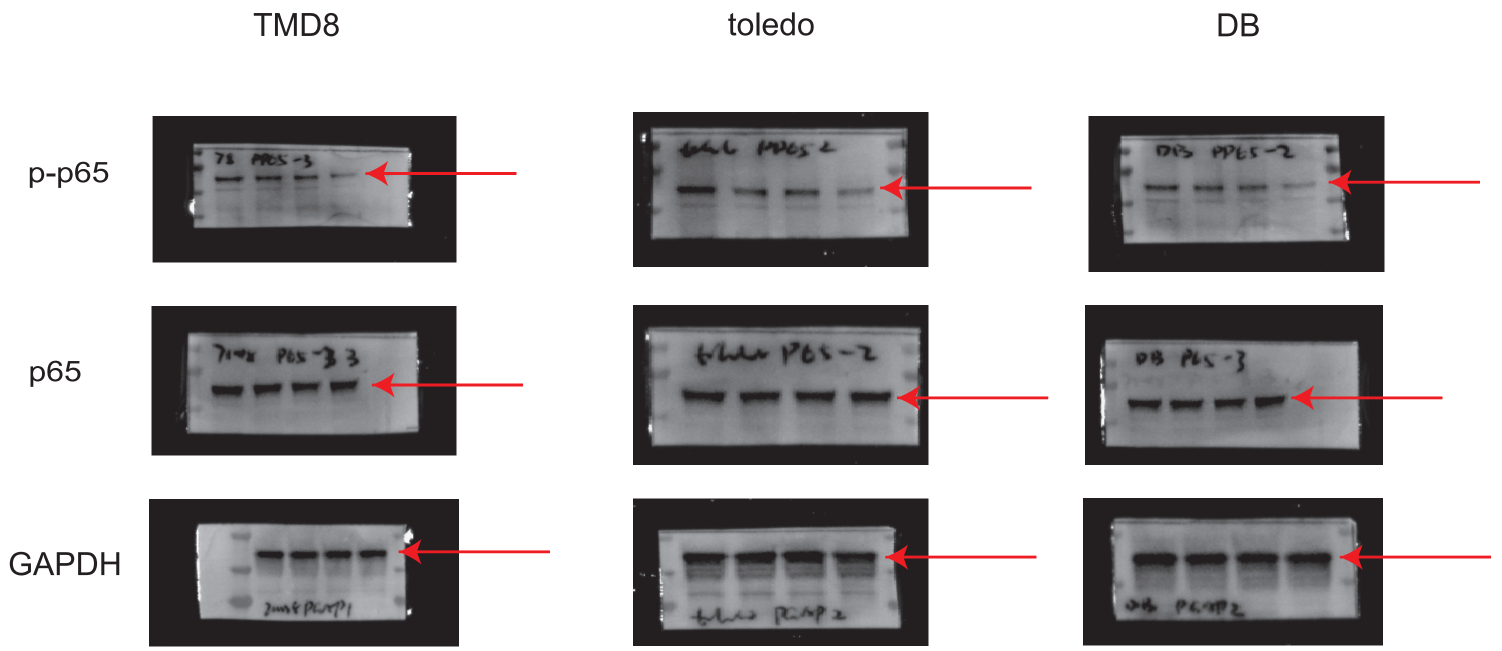

Figure 4E

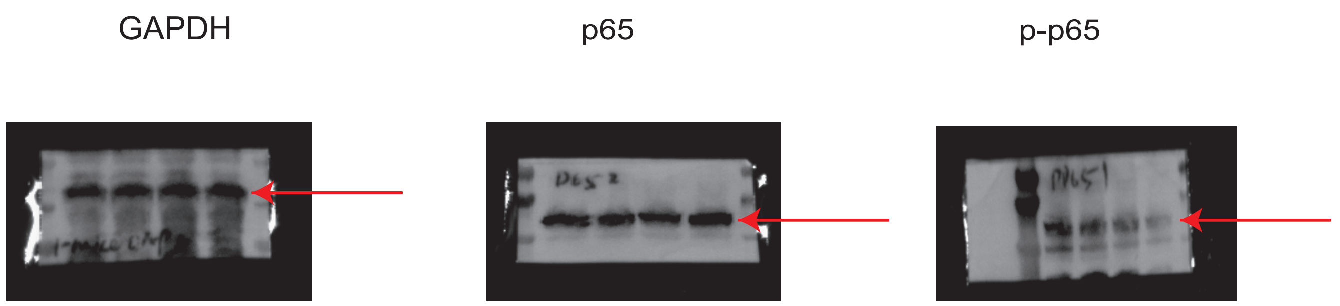

Figure 4G

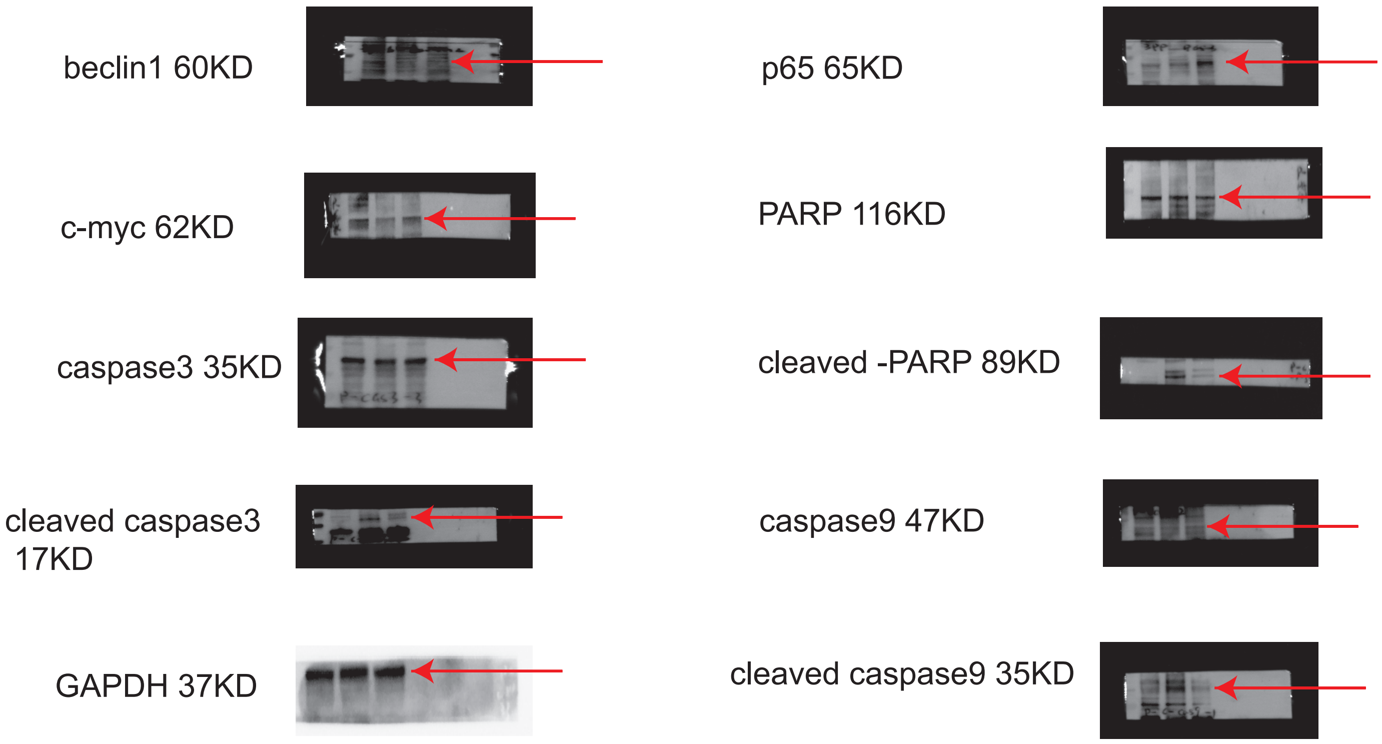

Figure 5A

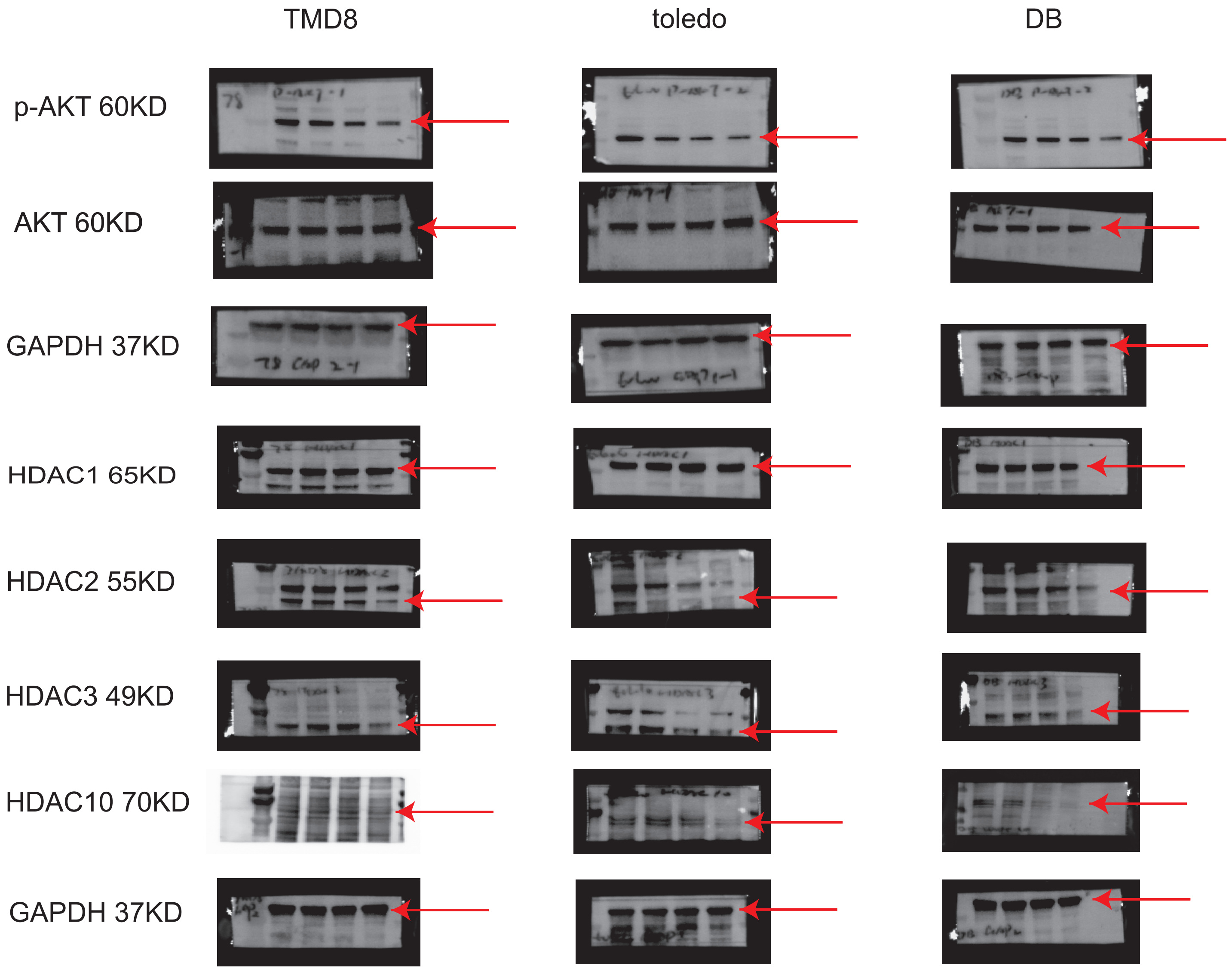

Figure 5B

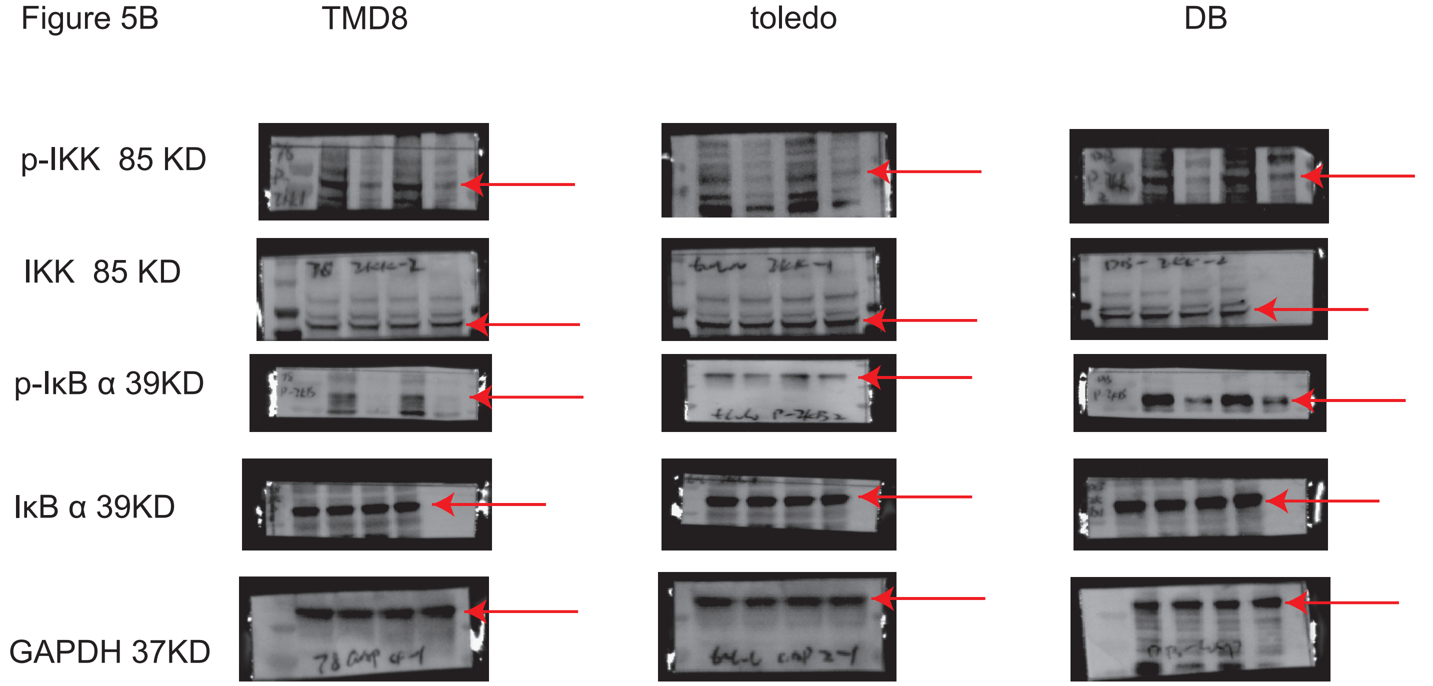

Figure 5C

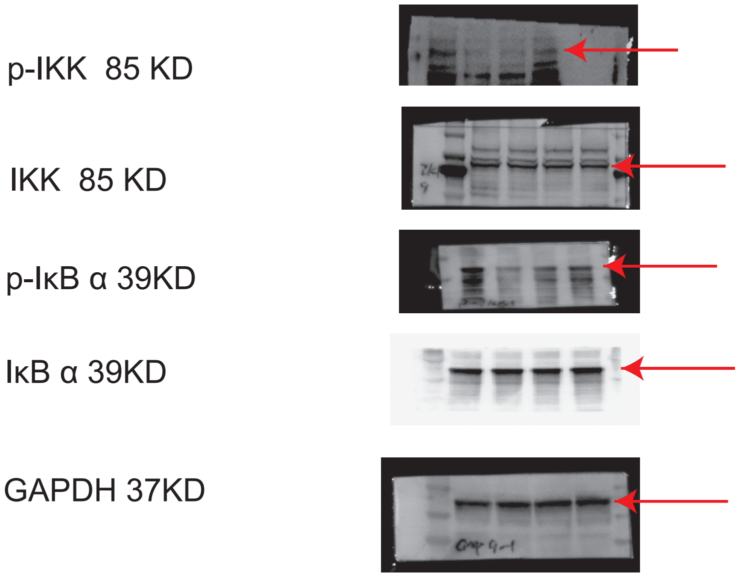

figure 5D

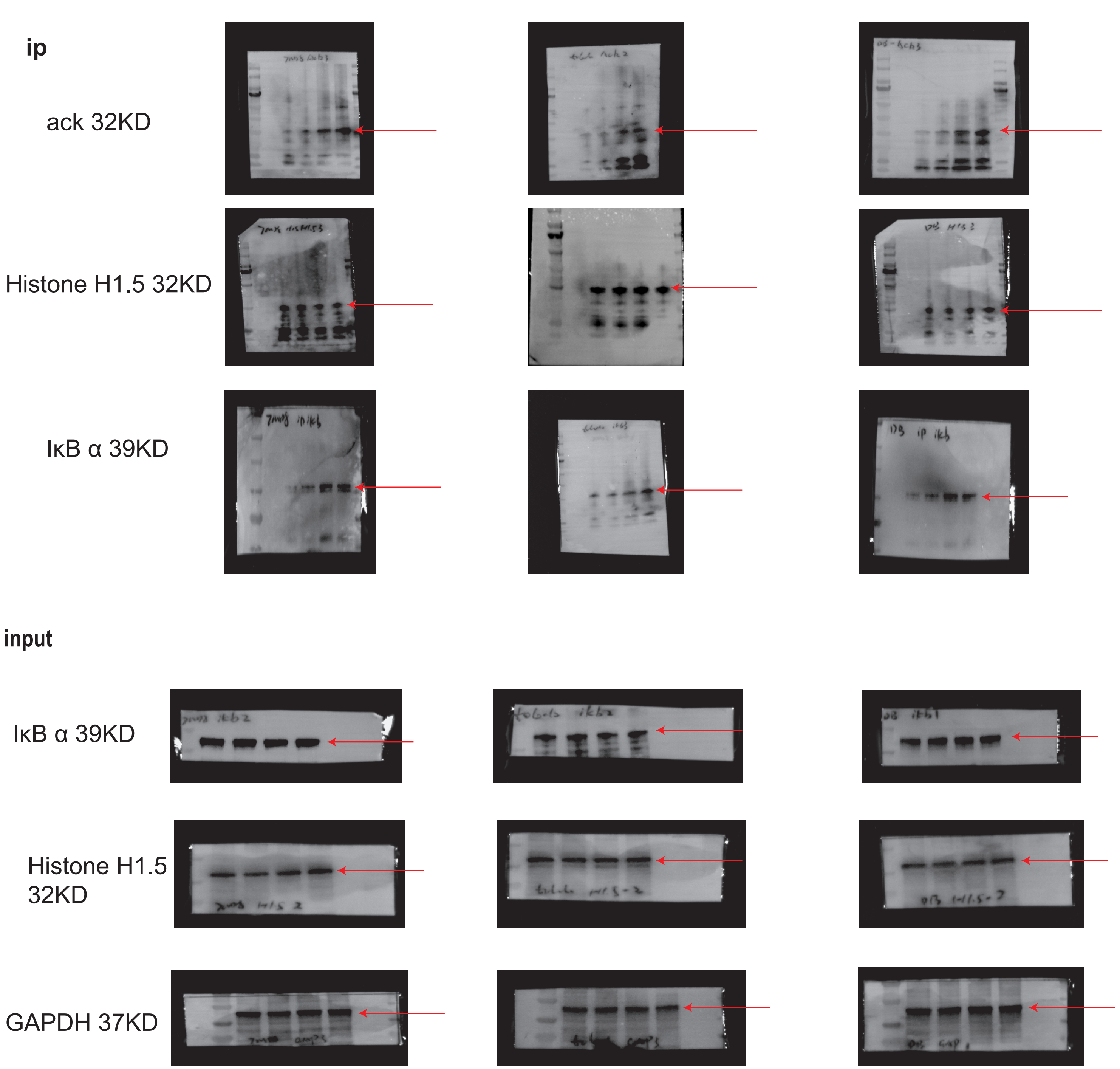

figure 5E

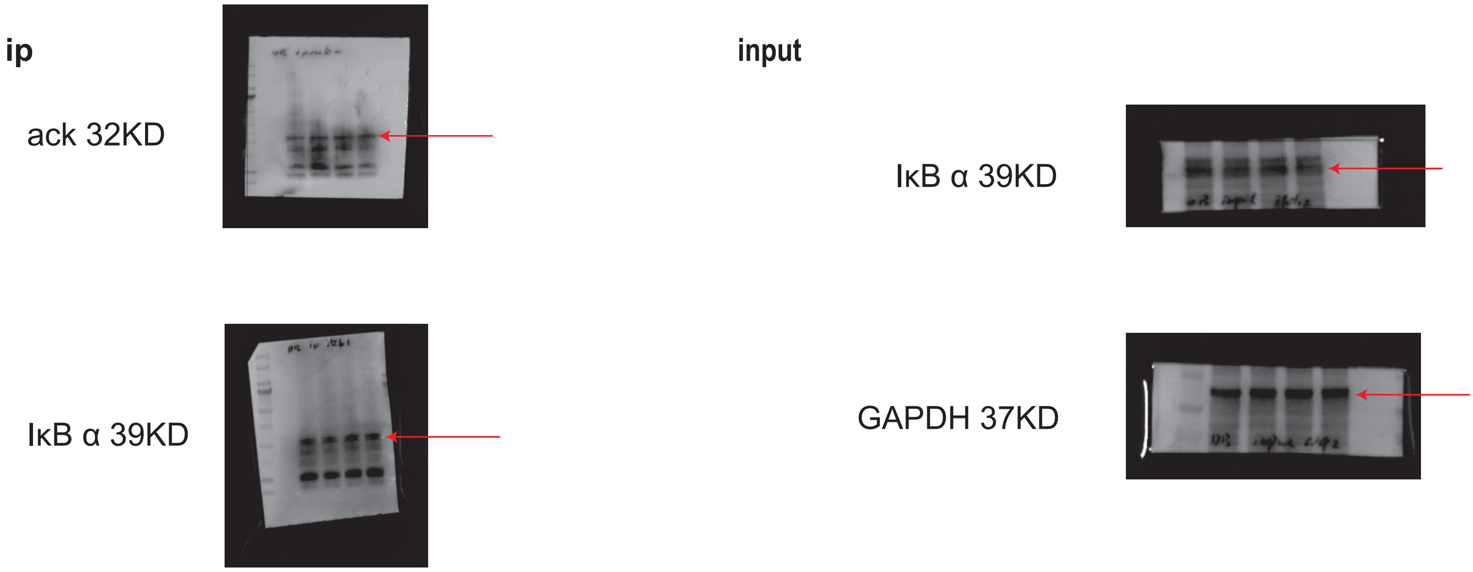

figure 6A

ip

ack 32KD

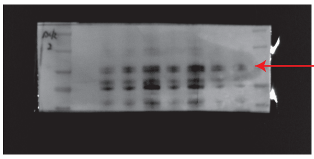

Histone H1.5 32KD

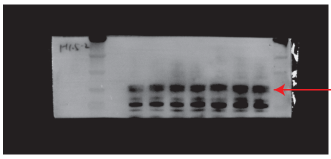

IκB α 39KD

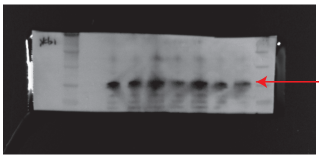

input

HDAC1 65KD

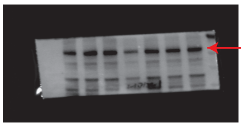

HDAC2 55KD

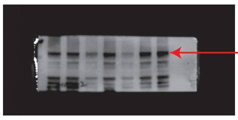

HDAC3 49KD

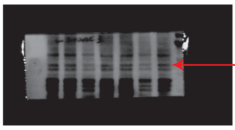

HDAC10 70KD

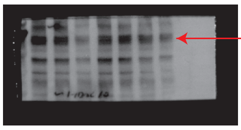

IκB α 39KD

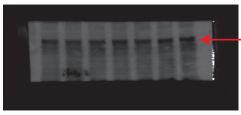

Histone H1.5 32KD

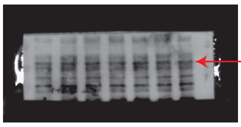

GAPDH 37KD

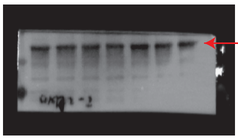

figure 6F

ip

ack 32KD

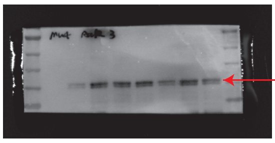

Histone H1.5 32KD

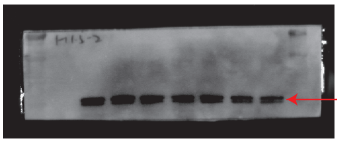

IκB α 39KD

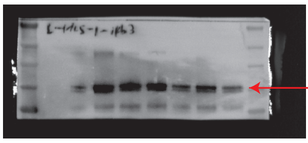

input

p65 65KD

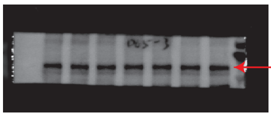

IκB α 39KD

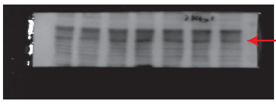

p-p65 65KD

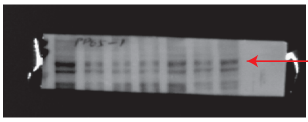

GAPDH 37KD

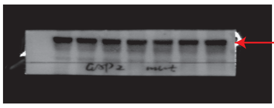

Histone H1.5 32KD

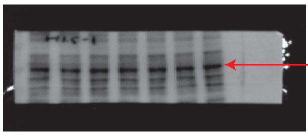

figure 7C

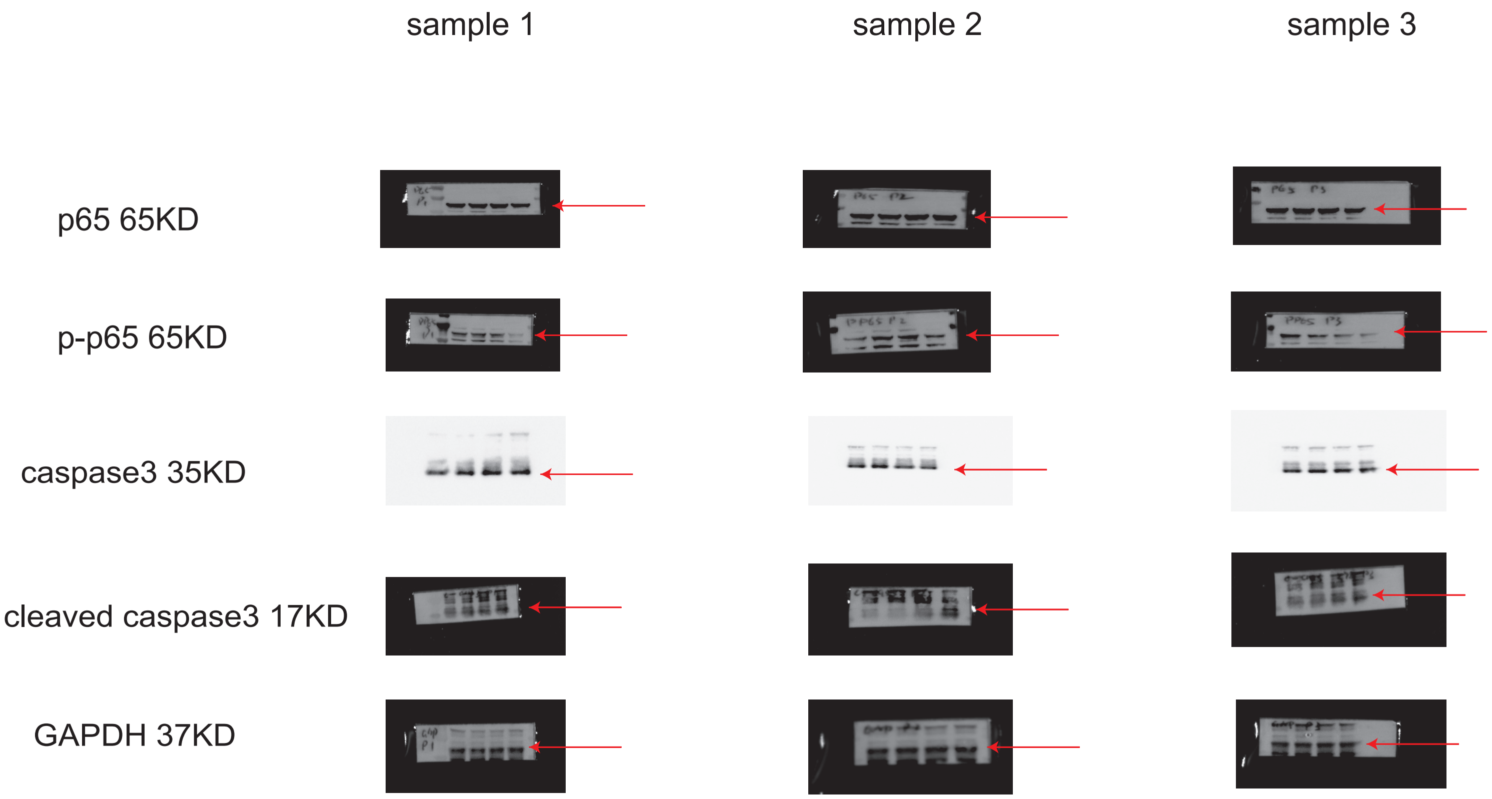
sample 1
sample 2
sample 3
p65 65KD
p-p65 65KD
caspase3 35KD
cleaved caspase3 17KD
GAPDH 37KD
